# Studies of All-*trans* Retinoic Acid Transport in the Eye

**DOI:** 10.1167/iovs.66.6.84

**Published:** 2025-06-30

**Authors:** Saptarshi Chatterjee, Ankana Roy, Jianshi Yu, Arthur Thomas Read, Melissa R. Bentley-Ford, Machelle T. Pardue, Maureen A. Kane, M. G. Finn, C. Ross Ethier

**Affiliations:** 1 Wallace H. Coulter Department of Biomedical Engineering, Georgia Institute of Technology & Emory University, Atlanta, Georgia, United States; 2 School of Chemistry and Biochemistry, Georgia Institute of Technology, Atlanta, Georgia, United States; 3 Department of Pharmaceutical Sciences, University of Maryland, Baltimore, Maryland, United States; 4 Department of Ophthalmology, Emory University School of Medicine, Atlanta, Georgia, United States; 5 Center for Visual and Neurocognitive Rehabilitation, Atlanta VA Healthcare System, Atlanta, Georgia, United States

**Keywords:** retinoic acid, retinoscleral signaling, myopia, transport phenomena, protein-ligand binding

## Abstract

**Purpose:**

Myopia incidence is increasing globally. All-*trans* retinoic acid (atRA) is important in myopigenic retinoscleral signaling, motivating research on its ocular transport. However, atRA's weak autofluorescence limits its direct visualization in tissues. Further, atRA is hydrophobic and must bind to protein carriers for transport. We assessed a fluorescent analog of atRA (LightOx 14, CAS:198696-03-6, referred as “floRA”), as an experimentally accessible atRA surrogate by: (i) evaluating its binding to carrier proteins and (ii) visualizing its distribution in ocular tissues.

**Methods:**

*Binding*: We assessed atRA-carrier protein binding using fluorescence quenching assays with bovine serum albumin (BSA), high density lipoprotein (HDL), apolipoprotein A-I (Apo A-I), and retinol binding protein 4 (RBP4). *Direct visualization*: Wild-type C57BL/6J mice were euthanized, their eyes were enucleated, and wedges containing sclera and choroid were incubated for specific durations in 50 µM floRA + BSA. The wedge centers were cryo-sectioned and counterstained for nuclei. Fluorescent micrographs were acquired and analyzed using ImageJ software.

**Results:**

Association constants (K_a_) for atRA and floRA binding to carrier proteins were similar and ranged from 2 to 13 × 10^5^ M^−1^, indicating nonspecific binding. floRA could be visualized in the sclera and choroid, yet showed significant spatial heterogeneity (enhanced fluorescence often colocalizing with nuclei).

**Conclusions:**

floRA is a reasonable surrogate for atRA binding to BSA, HDL, Apo A-I, and RBP4. Considering these proteins’ relative serum and extravascular abundances, and their similar binding affinity to atRA, we predict that serum albumin is an important atRA carrier. Use of floRA in whole tissue tracer studies shows promise but requires further refinement.

The global prevalence of myopia has markedly increased in recent decades.^1^^–^^3^ Severe myopia substantially enhances the risk of sight-threatening eye conditions, such as glaucoma, retinal detachment, and macular degeneration, establishing it as a leading risk factor for blindness worldwide.^4^ Myopia usually arises from excessive elongation of the eye along its optical axis and involves remodeling of the scleral extracellular matrix.^5^ Clinical and experimental investigations suggest that visual input through environmental stimuli is critical for the process that drives the overall axial length of the eye to come into balance with its refractive power, a process known as emmetropization.^6^ However, what is not understood is how these changes are processed by the retina and subsequently translated to the changes seen in the sclera, or how disruptions to these processes lead to refractive disorders such as myopia.^7^

It is widely recognized that scleral remodeling during myopigenesis involves a retinoscleral signaling cascade, which likely involves multiple signaling molecules, including all-*trans* retinoic acid (atRA).^8^^–^^11^ For example, it has been shown that orally delivered exogenous atRA in mice increases the concentration of atRA in several ocular tissue layers (including the sclera), induces myopia, and causes scleral remodeling,^12^ consistent with earlier studies in other species.^13^^–^^16^ Moreover, stromal cells in the inner choroid produce atRA^17^ and it is hypothesized that this is a key source of atRA during myopigenesis.

Despite the importance of understanding atRA-linked retinoscleral signaling pathways in myopigenesis, the mechanisms of atRA transport in relevant ocular tissues remain poorly understood. Because atRA is extremely hydrophobic,^18^ it must bind to one or more carriers for significant transport within aqueous environments.^19^ Previous studies identified some of these binding partners in different contexts,^20^^–^^25^ but uncertainty remains about the key binding partner(s) for atRA in the mammalian eye during myopigenesis. Further, quantitative, spatially resolved measurements of atRA concentrations in tissues are currently infeasible, in part because the weak autofluorescence of atRA limits its direct visualization in tissues using standard fluorescence microscopy. These factors greatly inhibit our understanding of atRA transport.

To better understand the role of atRA in myopigenic retinoscleral signaling, it would be very useful to be able to track the movement of atRA across the sclera and other relevant tissues. There exists a recently developed fluorescent analog of atRA (here, denoted as floRA; Fig. 1)^26^ that exhibits strong fluorescence when bound by a carrier protein but is non-fluorescent in aqueous environments.^26^ This allows one to image the spatial localization of floRA within cells and tissues, positioning it as a useful probe for investigating atRA transport in myopigenesis. Moreover, similar to atRA, floRA also possesses a hydrophobic structure^27^ and exhibits limited solubility in aqueous environments when not bound to a carrier protein. To confidently use floRA for this purpose, we must be sure that: (i) atRA and floRA have similar binding affinities to potential carrier proteins, and (ii) it is feasible to quantitatively track the location of a floRA-protein complex in tissue.

**Figure 1.**
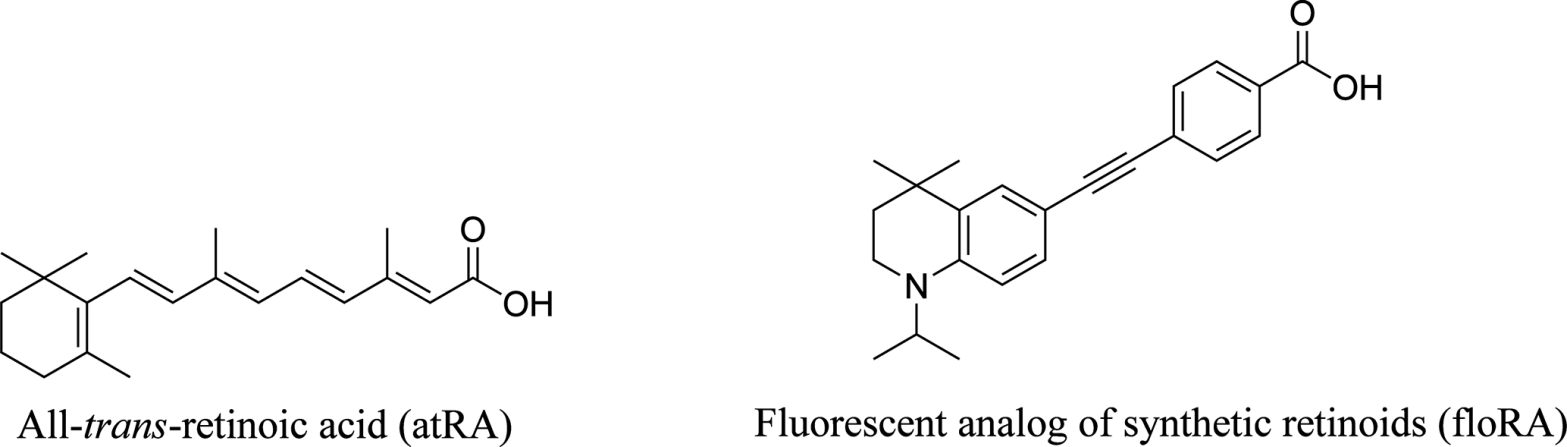
Chemical structures of all-*trans* retinoic acid (atRA) and its fluorescent analog (floRA).

Here, we investigated the binding affinity of selected proteins to atRA, focusing on their relevance to retinoscleral transport, and compared atRA and floRA binding to these proteins to evaluate the potential of floRA as a surrogate for atRA in tracer studies. Further, we conducted proof-of-concept experiments to visualize the presence of floRA in relevant ocular tissues from mice.

## Methods

### Reagent Specifications and Protein Production

Commercially available reagents were of reagent grade or better. atRA (≥98% pure, catalog no. R2625, CAS: 302-79-4) was purchased from Sigma-Aldrich (St. Louis, MO, USA) and was used without further purification. floRA (≥98% pure LightOx 14, CAS: 198696-03-6) was purchased from LightOx Limited (Newcastle upon Tyne, UK) in lyophilized solid form. Streptavidin Agarose resin (Pierce Streptavidin Agarose; catalog no. 20359) was purchased from Thermo Fisher Scientific (Waltham, MA, USA). This resin contains streptavidin recombinant protein crosslinked with 6% beaded agarose with particle size ranging from 45 to 165 µm. Sources of purchased proteins, and their concentrations as received, are given in Supplementary Table S1.

#### Cellular Retinoic Acid-Binding Protein 2

Mouse cellular retinoic acid-binding protein 2 (CRABP2) protein was expressed in One Shot BL21(DE3) Escherichia coli (*E. coli*; Thermo Fisher Scientific, Waltham, MA, USA) according to the manufacturer's instructions, using a plasmid custom synthesized by GenScript (Piscataway, NJ, USA). Protein purification was performed using Cytiva Glutathione Sepharose 4B (Millipore Sigma, Danvers, MA, USA), and the GST tag was removed by PreScission Protease (GenScript, Piscataway, NJ, USA). Buffer exchange of purified mouse CRABP2 used Amicon Ultra-15 Centrifugal Filter 3K Devices (Millipore Sigma, Danvers, MA, USA). Mouse CRABP2 protein concentration was determined from absorbance measurements at 280 nm using a calculated ε value of 19,480 M^−1^cm^−1^.^28^ Purity was assessed by subjecting 1 to 3 µg of purified protein to SDS-PAGE followed by determination of band intensity after Coomassie blue staining. The purified protein was then stored at −80°C in 1× phosphate-buffered saline (PBS; Thermo Fisher Scientific, Waltham, MA, USA) buffer solution.

#### Cellular Retinol-Binding Protein 1

Mouse cellular retinol-binding protein 1 (RBP1) was prepared according to the reported protocol.^29^^,^^30^ In brief, mouse RBP1 was expressed in BL 21 *E. coli* purchased from Sigma-Aldrich, utilizing plasmids sourced from Genecopia (Rockville, MD, USA), as per the manufacturer’s instructions. Purification procedures involved the use of a GE Healthcare GST bulk kit (GE Healthcare, Pittsburgh, PA, USA). Following expression, the GST tag was cleaved using Promega ProTEV protease (Promega, Madison, WI, USA). Subsequently, the removal of the protease was facilitated by GE Healthcare Ni resin. To ensure purity, the protein solution underwent a second pass through the GST column to separate the GST tag from the purified protein. The purified protein was then dialyzed and stored at −80°C in 20 mM KH_2_PO_4_ and 100 mM KCl buffer.

### Fluorescence and UV-Vis Spectroscopic Measurements

All spectra were acquired at room temperature. Fluorescence spectroscopic measurements were acquired with the sample solutions of volume approximately 700 µL in a 1.0 cm pathlength fluorescence quartz cuvette (FireflySci, Inc., Staten Island, NY, USA). Fluorescence spectra were recorded using a Horiba Jobin Yvon FL3-21 Fluorescence Spectrophotometer (Horiba Ltd., Kyoto, Japan) equipped with a 450-W Xe lamp, at a scan rate of 120 nm/min. The spectra were processed with FluorEssence (Horiba Ltd., Kyoto, Japan) software and plotted and analyzed using MATLAB 2023a (The MathWorks, Natick, MA, USA). In the fluorescence quenching experiments, the excitation wavelength was 280 nm, and the emission spectra were obtained in a wavelength range of 300 to 450 nm (protein-atRA binding) and 300 to 550 nm (protein-floRA binding). In fluorescence potentiation experiments with floRA, the excitation wavelength was 340 nm, and the emission spectra were obtained in a wavelength range of 400 to 600 nm.

UV-Vis absorbance spectra were obtained using an Evolution 220 UV-Vis spectrophotometer (Thermo Fisher Scientific, Waltham, MA, USA) with the sample solutions of volume approximately 700 µL in a 1.0 cm pathlength quartz cuvette (FireflySci, Inc., Staten Island, NY, USA). Prior to recording the spectra of the sample solutions, a baseline measurement was conducted using the same PBS buffer solution used throughout the experiment. This baseline correction was then automatically applied to each spectrum by using default software settings. The spectra were processed with INSIGHT software (Thermo Fisher Scientific, Waltham, MA, USA), plotted, and further analyzed with MATLAB. The measurements were conducted across the UV-Vis region. However, we here focus on presenting spectra over the range 250 to 550 nm, because no significant spectral features were observed outside this range.

For comparative binding experiments with atRA, Apo A-I, and biotinylated BSA, fluorescent spectra were recorded utilizing a FlexStation3 (Molecular Devices, LLC., San Jose, CA, USA) microplate reader, processed with SoftMax Pro 5.4.6 (Molecular Devices, LLC., San Jose, CA, USA) software, plotted, and analyzed using MATLAB. Each experiment utilized 40 µL of sample solution.

### Determination of Protein-atRA and Protein-floRA Association Constants and Stoichiometry From Fluorescence Quenching Data

To determine the association constants of protein-atRA/floRA binding, we utilized the quenching of the proteins’ intrinsic tryptophan fluorescence through complexation between the carrier protein and atRA/floRA (Supplementary Fig. S1). The protein concentrations ranged from 1 to 6 µM and the amounts of atRA/floRA titrated ranged from 0 to 25 µM. Full details are provided in Supplementary Table S2. Upon addition of atRA/floRA to the protein solution, the sample was mixed well and incubated on ice for 15 to 20 minutes before measurement of fluorescence spectra. The results from fluorescence measurements were fitted to standard models (Stern-Volmer, modified Stern-Volmer,^22^^–^^24^^,^^31^^,^^32^ and quadratic^25^^,^^29^^,^^30^) to estimate the association constants of retinoid–protein complexation, as described in detail in the Supplementary Material. In the main text, we report values of the association constant based on quadratic fitting, because the quadratic model can deal with nonlinear trends in fluorescence quenching data at high concentrations. In the Table, we show association constant values based on all fitting approaches to allow comparison with previous literature reports. Detailed results from other fitting approaches are shown in the Supplementary Material.

**Table.** Association Constant Values (K_a_, Mean ± SD) Estimated From Fluorescence Quenching Experiments With atRA and floRA, as Obtained by Different Fitting Approaches (see Methods)

		Association Constant			
			mSV Model		
Protein	Ligand	SV Model K_a_ (×10^5^ M^−1^)	K_a_ (×10^5^ M^−1^)	n	Quadratic Model K_a_ (×10^5^ M^−1^)
BSA	atRA	1.6 ± 0.2	1.4 ± 0.4	1.1 ± 0.3	2.4 ± 0.3
	floRA	1.3 ± 0.1	1.0 ± 0.4	1.3 ± 0.4	2.1 ± 0.4
HDL	atRA	6.4 ± 0.3	6.9 ± 0.9	1.0 ± 0.1	10.1 ± 5.4
	floRA	5.9 ± 0.2	3.4 ± 0.0	1.4 ± 0.0	13.2 ± 1.1
Apo A-I	atRA	1.2 ± 0.2	1.3 ± 0.4	1.0 ± 0.3	6.3 ± 0.3
	floRA	1.7 ± 0.5	1.5 ± 0.5	1.1 ± 0.2	7.9 ± 0.2
RBP4	atRA	1.5 ± 0.2	1.7 ± 0.8	1.0 ± 0.3	6.6 ± 0.2
	floRA	0.3 ± 0.0	0.6 ± 0.1	0.7 ± 0.1	4.3 ± 0.2
CRABP2	atRA	–	–	–	784 ± 634
	floRA	–	–	–	669 ± 92
RBP1	atRA	–	–	–	–
	floRA	1.5 ± 0.3	1.4 ± 1.3	1.2 ± 0.4	7.9 ± 0.4

SV, Stern-Volmer; mSV, modified Stern-Volmer.

Dashes indicate conditions where measurements/K_a_ estimations were not made.

### Determination of Binding Constants From Fluorescence Potentiation Experiments With floRA

Fluorescence emission of floRA in an aqueous solution is severely quenched, but this fluorescence is increased in a concentration-dependent manner when floRA is bound to a hydrophobic binding pocket of a protein in an aqueous solution.^26^ We exploited this behavior and implemented an experimental approach, denoted as “fluorescence potentiation experiments” to estimate the floRA-protein association constant. In these experiments, the floRA concentration was fixed, whereas the protein concentration was gradually increased causing the fluorescence emission intensity to reach a plateau at a certain protein concentration threshold.^26^ floRA concentration ranged from 0.025 to 0.05 µM, and the protein concentrations titrated into the floRA solution ranged from 0 to 35 µM. Full details are provided in Supplementary Table S3. Upon the addition of floRA to the protein solution, the sample was mixed well followed by incubation on ice for 15 to 20 minutes before fluorescence measurement. We fitted the Hill Equation (taking the Hill coefficient as unity, i.e. ignoring the potential effect of cooperativity in protein-ligand binding) to the fluorescence data to evaluate the binding constant^33^ using the “fit” function in MATLAB. The Hill equation (Equation 1) is expressed in relation to the fluorescence data as:

(1) $$\begin{array}{c} F/F_{max}=\frac{\left[P_{T}\right]}{\left[P_{T}\right]+K_{D}} \end{array}$$

where [P_T_] is the total protein concentration (µM), *F_max_* represents the fluorescence signal obtained at the spectral maxima once the saturation plateau is attained, and *F* is the fluorescence intensity at protein concentration [*P_T_*]. *K_D_* represents the dissociation constant (estimated from the fitting), the inverse of which is taken as the association constant *K_a_* (M^−1^).

### Protein-Ligand Complex Isolation Protocol

A mixture of 1 mM floRA in ethanol (0.08 mL) and 0.1 mM BSA in PBS (1.4 mL) was passed through a PD-10 desalting column (freshly equilibrated with PBS buffer), with an additional 1.0 mL of PBS added concurrently. The initial eluate (approximately 2.5 mL) was discarded, and the BSA-floRA complex was isolated by adding a total of 4.0 mL of PBS in 0.5 mL increments, with eluate collected into 8 separate Eppendorf tubes. UV-Vis absorbance revealed the presence of BSA-floRA in the first three eluate fractions, which were combined and used directly for incubation with mouse scleral tissue, as described in the section titled “Visualization of floRA in Ocular Tissues” below.

### Competitive Binding Experiments With atRA, Apo A-I, and Biotinylated BSA

Polypropylene chromatography columns were packed with 2 mL of streptavidin-agarose resin, followed by equilibration with PBS (2 × 10 mL). Biotinylated BSA (50 µM, 1 mL) in PBS was passed through the column. The eluate was collected and passed through the column twice more, after which no UV-visible absorbance was detected in the eluate indicating complete adsorption of the biotinylated BSA by the streptavidin resin. A solution of atRA (50 µM, 1 mL) in PBS and ≤5% ethanol (EtOH) was then passed through the column. The eluate was then collected and analyzed, revealing no detectable absorbance for either biotinylated BSA or atRA, indicating successful BSA-atRA binding.

To investigate whether atRA selectively favors one potential carrier over another, a solution of 50 µM Apo A-I and 50 µM atRA was passed through a streptavidin-biotinylated BSA column (prepared as above). The eluate was then collected and circulated through the column four times, and the UV-Vis absorbance of each eluate was recorded. Subsequently, approximately 10% to 15% ethanol was added to the fourth eluate. This combined solution was passed through the column and collected for spectroscopic measurements.

### Visualization of floRA in Ocular Tissues

All animal procedures adhered to the ARVO Statement for the Use of Animals in Ophthalmic and Vision Research, and to a protocol approved by the Institutional Animal Care and Use Committee at Emory University. Male and female C57BL/6J mice were procured from Jackson Laboratory (Bar Harbor, Maine, USA) and housed in the animal facility at Emory University on a 12-hour light-dark cycle. Mice approximately 4 to 5 weeks of age were euthanized by cervical dislocation. Their eyes were then enucleated, placed in 0.1 M (1×) PBS, dissected to carefully remove extraocular muscle, fat, and conjunctiva, and hemisected at the corneoscleral junction. The anterior segment and intraocular tissues were removed to leave scleral shells with adherent choroid. Each posterior shell was cut into quadrants, which were further cut into pie-shaped wedges. These were incubated for various durations (5 minutes, 10 minutes, 30 minutes, 60 minutes, 90 minutes, and 180 minutes) at 4°C in a BSA-floRA solution (approximately 50 µM floRA-BSA complex in PBS and ≤5% EtOH). Prior to incubation, this solution underwent a protein-ligand complex isolation protocol, as described above.

Tissue wedges were embedded in OCT compound (Fisher HealthCare, Houston, TX, USA), snap-frozen and stored at −80°C. Sections, 10 µm thick, were cut on a cryostat and collected on glass slides. To reduce diffusion of floRA out of the tissue sections, solution changes and washes were eliminated. Specifically, to stain nuclei, we prepared a mixture of ProLong Gold media and Sytox Orange (both from Thermo Fisher Scientific, Waltham, MA, USA, diluted 100:1, respectively) that was vortexed briefly, and then centrifuged. After collected sections were allowed to dry for 10 minutes, the slides were mounted using the above mixture and cover-slipped, omitting the permeabilization step and washes. The slides were allowed to cure overnight at 4°C, and then imaged with a Leica DM6 fluorescent microscope (Leica Microsystems, Wetzlar, Germany) equipped with the appropriate filter cubes. Because floRA was found to be highly sensitive to bleaching and fading, illumination was strictly minimized in terms of exposure time and intensity, and imaging for each experiment was accomplished within 2 hours. Brightfield and fluorescent (floRA and Sytox Orange channels) images were acquired (16 bit, 2048 × 2048 pixels, and 6.2 pixels/micron) and stored for later analysis.

Fluorescent images showed significant local spatial heterogeneity in floRA intensity in both the sclera and choroid, making extraction of reliable spatially resolved fluorescence intensity profiles infeasible. Instead, we quantified average floRA fluorescence intensity within a large region of the sclera at different time points, thereby seeking to reduce the effects of spatial heterogeneity by spatial averaging. Specifically, by simultaneous inspection of paired brightfield and floRA fluorescence images in Fiji version 1.54k,^34^ we manually drew curves along the outer scleral margin and the scleral-choroidal interface. We then connected these curves by straight lines across the sclera (Supplementary Fig. S2), to create a quadrilaterally shaped scleral region. The mean fluorescent intensity (after correction for background fluorescence) within this region and the area of this region were obtained in Fiji. This was repeated on four sections per time point (technical replicates) for two eyes (biological replicates). The area of the analyzed scleral region varied somewhat from one section to another, but was typically 8700 square microns, and scleral thickness was typically approximately 40 microns.

## Results

### Overview of atRA/floRA Protein Binding Experiments

The suitability of floRA as a fluorescent atRA analog in tracer studies was studied by characterizing the binding of both atRA and floRA to the following carrier proteins using protein-dependent changes in fluorescence spectra: bovine serum albumin (BSA), high-density lipoprotein (HDL), apolipoprotein A-I (Apo A-I, a part of the HDL complex^35^), and retinol binding protein 4 (RBP4). We also used CRABP2 and cellular retinol-binding protein 1 (RBP1) as positive and negative controls, respectively, due to their known binding affinities with atRA.^25^^,^^29^^,^^36^^,^^37^ We chose BSA and HDL because they are potential carriers of atRA through the extracellular space, including within the bloodstream. Apo A-I has been implicated as an atRA binding protein in the eye in myopia,^20^ wheras RBP4, a known chaperone for all-trans-retinol,^38^^–^^40^ which is known to bind retinol and atRA with similar binding affinity^40^^,^^41^ may facilitate atRA transport before cellular uptake. Uptake of retinoic acid has been observed in pigment epithelial cells in vitro through dissociation of retinoic acid-RBP complex.^42^

All the above proteins have intrinsic tryptophan fluorescence that can be quenched upon atRA binding to the protein.^20^^,^^24^^,^^25^ We made use of this phenomenon to evaluate the binding affinities of atRA to these potential carrier proteins. Further, because atRA can bind to multiple carrier proteins,^20^^–^^25^ we also investigated atRA's propensity to switch from one protein carrier to another.

### Control Experiments With CRABP2 and RBP1

Previous research has quantified the strong affinity of both atRA and floRA for CRABP2.^25^^,^^26^^,^^37^ Thus, we chose CRABP2 as a positive control for our fluorometric titration experiments. The expected quenching of CRABP2 tryptophan fluorescence was observed upon the addition of atRA or floRA (Supplementary Fig. S3), with no noticeable alterations detected in other spectroscopic parameters. We also observed an additional emission peak around 463 nm (upon excitation at 280 nm; Supplementary Fig. S4) due to the intrinsic fluorescence of the floRA in the presence of CRABP2 (see Supplementary Fig. S3C). Association constants of atRA and floRA with CRABP2 determined using these fluorescence signals were closely comparable (see the Table, Supplementary Table S4) and aligned well with the lower range of values documented in the literature (for atRA-CRABP2, 700–5000 × 10^5^ M^−1^^25^^,^^36^^,^^37^; for floRA-CRABP2, 300 × 10^5^ M^−1^).^26^

Conversely, previous studies have shown that atRA does not bind to RBP1,^29^^,^^30^ suggesting its utility as a negative control. In fluorescence quenching experiments, we found that RBP1 tryptophan emission intensity did not change as a function of atRA concentration, confirming its expected lack of binding (Supplementary Figs. S5A, S5B). However, similar experiments using floRA instead of atRA showed quenching of RBP1 fluorescence by floRA in a concentration-dependent manner (Supplementary Figs. S5C–S5F), indicating dissimilarities between atRA and floRA when interacting with RBP1. Quenching of RBP1 fluorescence by floRA allowed us to estimate an apparent association constant of 7.9 ± 0.4 × 10^5^ M^−1^ (see the Table).

### atRA and floRA Show Comparable Binding Affinities to BSA, HDL, and Apo A-I

**Figure 2.**
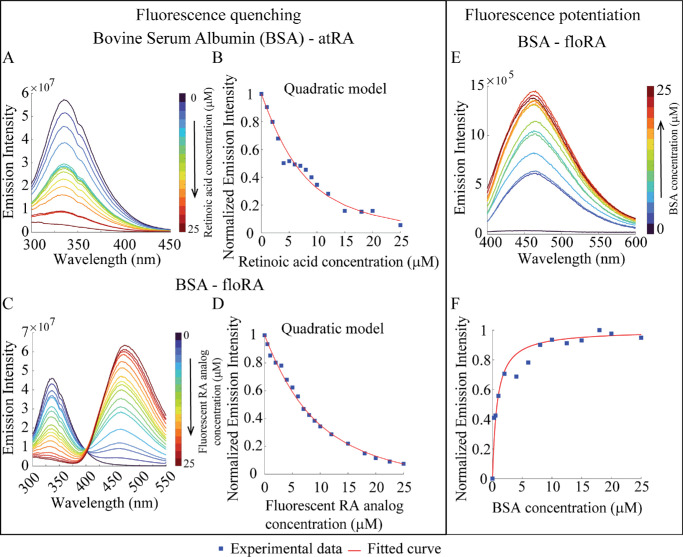
**Fluorometric investigations of BSA-retinoic acid binding.** (**A**, **C**) Fluorescence emission spectra of 6 µM BSA in the presence of atRA or floRA, respectively (0–25 µM). The quenching of BSA fluorescence (336 nm) with increasing concentrations of atRA or floRA is evident. (**B**, **D**) Quadratic model fits to the peak emission intensities at 336 nm from the spectra in panels **A** and **C**. (**E**) Fluorescent emission spectra of floRA (0.05 µM) in the presence of BSA (0–25 µM); increase in peak intensity indicates increasing amounts of floRA bound by the protein. (**F**) Hill curve fit of fluorescence intensity (461 nm) in panel **E**. Panels **A** to **F** show typical results from three technical replicates.

The binding of atRA to BSA was similarly quantified using BSA's intrinsic tryptophan fluorescence quenching (λ_em_ = 336 nm and λ_ex_ = 280 nm) upon ligand exposure^22^^–^^24^^,^^43^ (Fig. 2A, see Supplementary Figs. S1, S6A, S6B). atRA alone is non-fluorescent in water and the addition of atRA to BSA induces no other discernible spectroscopic alterations (see Figs. 2A, 2B, Supplementary Figs. S7A, S7B). The addition of floRA to BSA caused a similar loss of BSA tryptophan fluorescence emission in a concentration-dependent manner (see Figs. 2C, 2D, Supplementary Figs. S7C, S7D), along with the appearance of an additional emission peak at 461 nm (λ_ex_ = 280 nm) due to the intrinsic fluorescence of the floRA in the presence of BSA (see Fig. 2C). floRA alone, in the absence of BSA, remained non-fluorescent in an aqueous environment (Supplementary Fig. S8).

Using tryptophan fluorescence data, the association constants for BSA-atRA and BSA-floRA binding were found to be 2.4 ± 0.3 × 10^5^ M^−1^ and 2.1 ± 0.4 × 10^5^ M^−1^, respectively (see the Table), consistent with previous reports for albumin-atRA binding (3–23 × 10^5^ M^−1^^22^^–^^24^^,^^44^). The concentration-dependent appearance of floRA fluorescence in complexation with BSA (see Figs. 2E, 2F) provided an estimated association constant of 11.8 ± 1.2 × 10^5^ M^−1^ (Supplementary Table S4). Although somewhat different than the tryptophan-derived value, the binding affinities of atRA and floRA to BSA are of the same order of magnitude, enhancing confidence in floRA's potential role as a probe for investigating BSA-mediated atRA transport.

**Figure 3.**
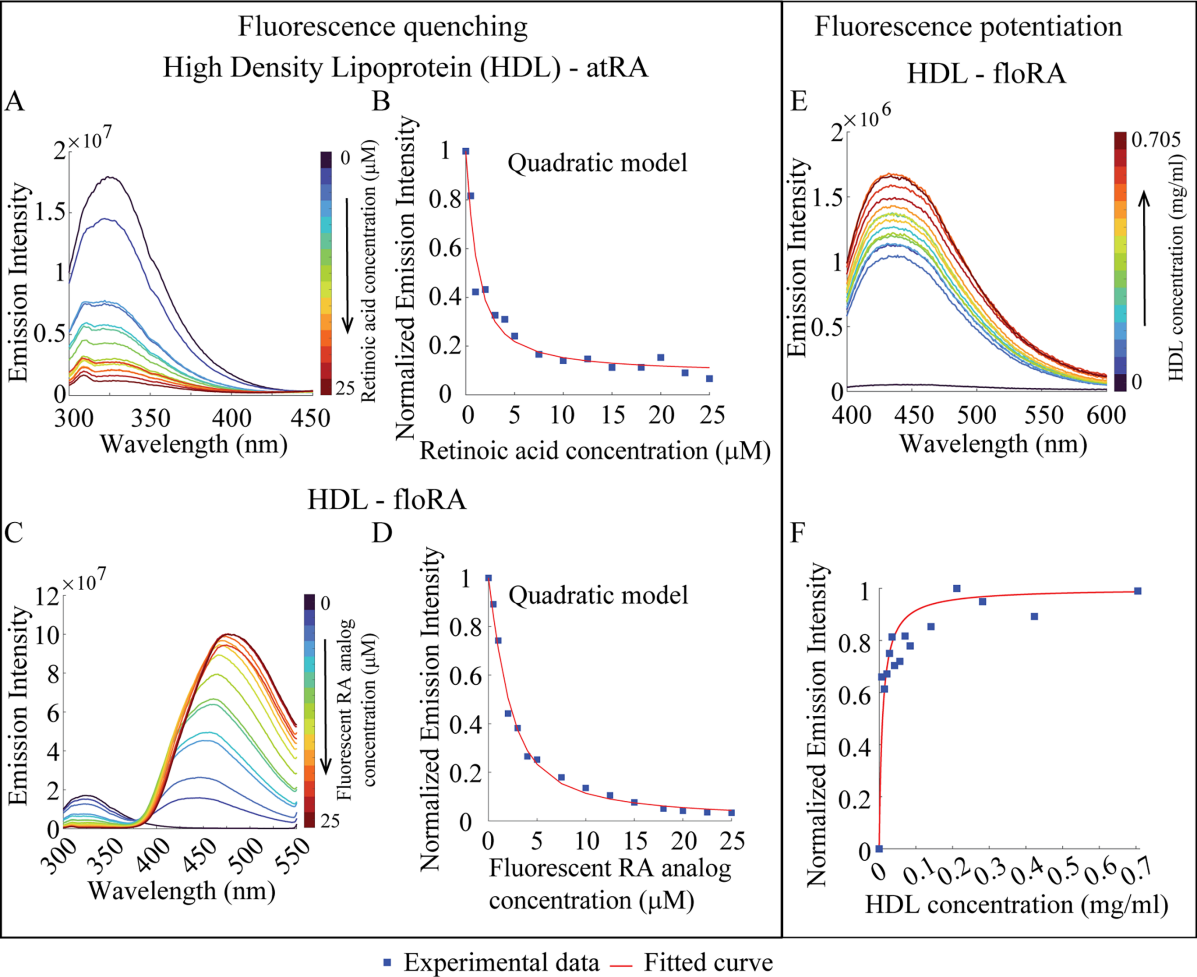
**Fluorometric investigations of HDL-retinoic acid binding.** (**A**, **C**) Fluorescence emission spectra of HDL (0.028 mg/mL) in the presence of atRA or floRA, respectively (0–25 µM). The quenching of HDL fluorescence (321 nm) with increasing concentrations of atRA or floRA is evident. (**B**, **D**) Quadratic model fits to the peak emission intensities at 321 nm from the spectra in panels **A** and **C**. (**E**) Fluorescent emission spectra of floRA (0.05 µM) in the presence of HDL (0–0.7 mg/mL); the increase in peak intensity indicates increasing amounts of floRA bound to HDL. (**F**) Hill curve fit of fluorescence intensity (437 nm) in panel **E**. Panels **A** to **F** show typical results from three technical replicates.

**Figure 4.**
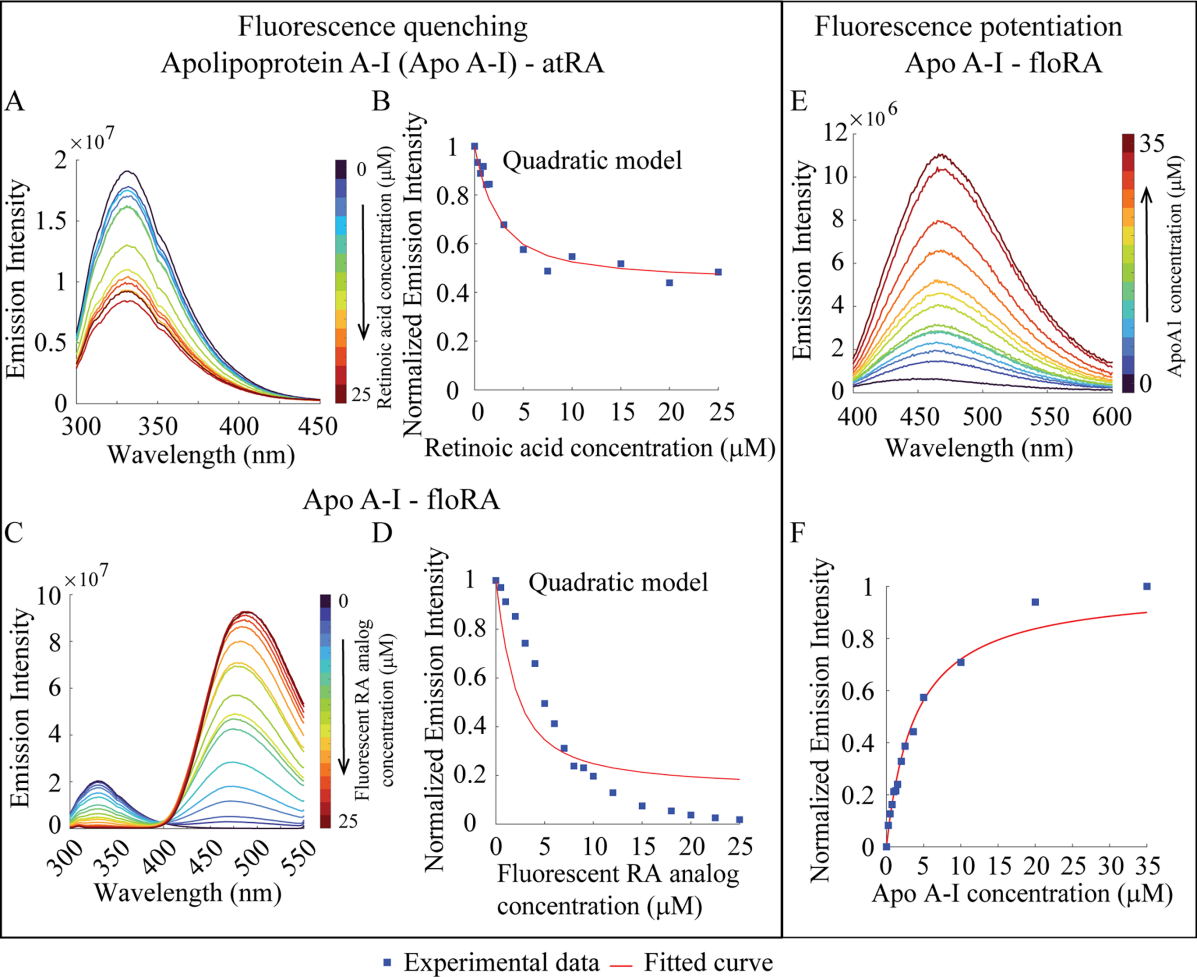
**Fluorometric investigations of Apo A–I-retinoic acid binding.** (**A**, **C**) Fluorescence emission spectra of Apo A-I (1.2 µM) in the presence of atRA or floRA, respectively (0–25 µM). The quenching of Apo A-I fluorescence (332 nm) with increasing concentrations of atRA or floRA is evident. (**B**, **D**) Quadratic model fits to the peak emission intensities at 332 nm from the spectra in panels **A** and **C**. (**E**) Fluorescent emission spectra of floRA (0.05 µM) in the presence of Apo A-I (0–35 µM); the increase in peak intensity indicates increasing amounts of floRA bound to the protein. (**F**) Hill curve fit of the fluorescence intensity (467 nm) in panel **E**. Panels **A** to **F** show typical results from three technical replicates.

Previous work has indicated that Apo A-I, a major constituent of HDL, may be an important carrier of atRA in myopigenesis.^20^ Both tryptophan fluorescence quenching and floRA fluorescence potentiation were observed upon the addition of atRA and floRA to HDL and Apo A-I in a similar manner to BSA (Figs. 3, 4; see also supporting data in Supplementary Figs. S9, S10, Supplementary Tables S2–S4). As was the case with BSA, fitting of the fluorescence quenching data showed that HDL-atRA (10.1 ± 5.4 × 10^5^ M^−1^) and HDL-floRA (13.2 ± 1.1 × 10^5^ M^−1^) association constants were comparable (see the Table), as was the value determined using HDL-floRA fluorescence potentiation (10.3 ± 3.5 × 10^5^ M^−1^; see Supplementary Table S4). Of note, because the HDL solution we used contains multiple proteins including Apo A-I and Apo A-II and their relative fraction was unknown, we determined the effective protein concentration within HDL by treating this concentration as a free parameter in the quadratic model fitting, allowing us to convert the known HDL concentration (in terms of mg/mL) into µM. The details of this analysis are provided in the Supplementary Material.

Apo A-I binding led to generally similar fluorescence behavior as HDL,^20^ but we observed disagreement between data and quadratic model fitting in Figure 4D. To ensure that the fitted curve was not trapped in a local minimum, we used several approaches: we first implemented simulated annealing (simulannealbnd) in MATLAB to refine the initial guess for lsqcurvefit. Because simulated annealing is stochastic, we ran the optimization multiple times with different initial conditions, but this did not improve the fitting quality. We then used another global optimization method, the Genetic Algorithm (ga), which does not require a single initial guess but instead explores the parameter space using a population-based approach. However, this too did not enhance the fit of the curve presented in Figure 4D. Because neither simulannealbnd nor ga improved the fitting quality, it is likely that the model itself cannot adequately capture the data trends in Figure 4D. The discrepancy may stem from the molecular interactions between floRA and Apo A-I affecting the energy transfer during the tryptophan fluorescence quenching. Whereas all data fitting models assume static quenching, in which the quencher forms a non-fluorescent ground state complex, the existence of dynamic quenching cannot be ruled out. Further, it is documented that Apo A-I undergoes self-assembly and form Apo A-I globular condensates.^45^ Previous studies have shown that protein aggregation can induce self-quenching of fluorescence suggesting that the aggregation induced self-quenching could also contribute to the observed discrepancy.^46^^,^^47^ Therefore, although the fitted association constants for Apo A-I-atRA binding (6.3 ± 0.3 × 10^5^ M^−1^) and Apo A-I-floRA binding (7.9 ± 0.2 × 10^5^ M^−1^) were similar (see the Table), there may be some differences in floRA-Apo A-I binding versus atRA-Apo A-I binding. The binding constant obtained from fluorescence potentiation of floRA upon Apo A-I binding (2.5 ± 0.1 × 10^5^ M^−1^; see Figs. 4E, 4F, Supplementary Table S4) was again similar to that obtained by tryptophan fluorescence quenching.

### atRA and floRA Binding to RBP4

**Figure 5.**
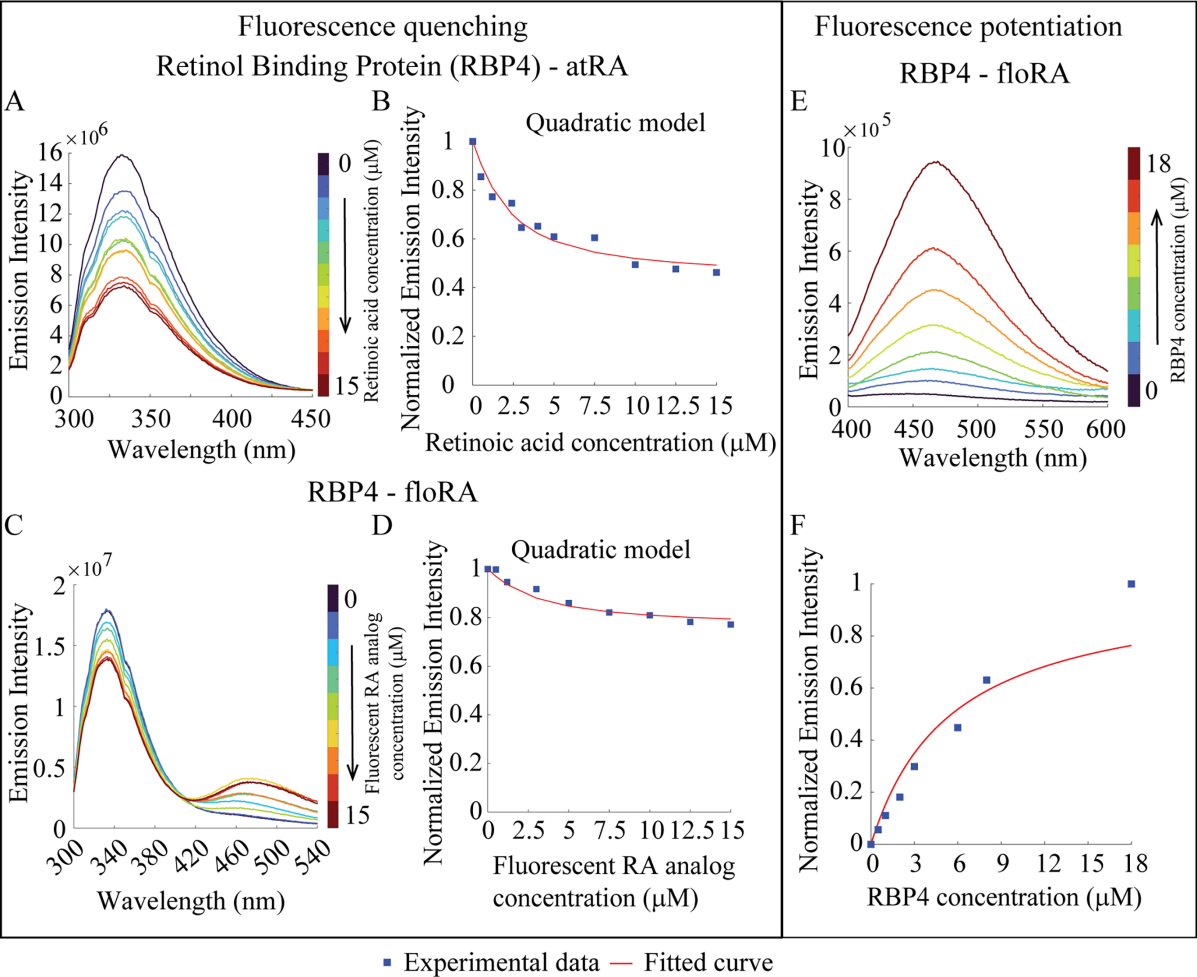
**Fluorometric investigations of RBP4-retinoic acid binding.** (**A**, **C**) Fluorescence emission spectra of RBP4 (1.2 µM) in the presence of atRA or floRA, respectively (0–15 µM). The quenching of RBP4 fluorescence (334 nm) with increasing concentrations of atRA or floRA is evident. (**B**, **D**) Quadratic model fits to the peak emission intensities at 334 nm from the spectra in panels **A** and **C**. (**E**) Fluorescent emission spectra of floRA (0.025 µM) RBP4 (0–18 µM); the increase in peak intensity indicates increasing amounts of RBP4 bound to the protein. (**F**) Hill curve fit of the fluorescence intensity (466 nm) in panel **E**. Panels **A** to **F** show typical results from three technical replicates.

It is known that retinol (ROL) circulating in the bloodstream is transported by RBP4.^48^^–^^51^ We characterized RBP4 binding to atRA^40^^,^^41^ and floRA by examining the quenching of RBP4 tryptophan emission upon binding with atRA and floRA (Figs. 5A–D, Supplementary Fig. S11, see Supplementary Table S2). The derived association constants for RBP4-atRA and RBP4-floRA were again similar, 6.6 ± 0.2 × 10^5^ M^−1^ and 4.3 ± 0.2 × 10^5^ M^−1^, respectively (see the Table). Interestingly, even with higher concentrations of the quencher floRA, the peak tryptophan fluorescent emission intensity was reduced by only approximately 20% (see Fig. 5D), suggesting some differences in the binding geometries of atRA versus floRA to RBP4. This was supported by the lack of saturated fluorescence turn-on of floRA by interaction with RBP4 (Figs. 5E, 5F).

### Competition Between Immobilized and Solution-Phase Binding Proteins for atRA

**Figure 6.**
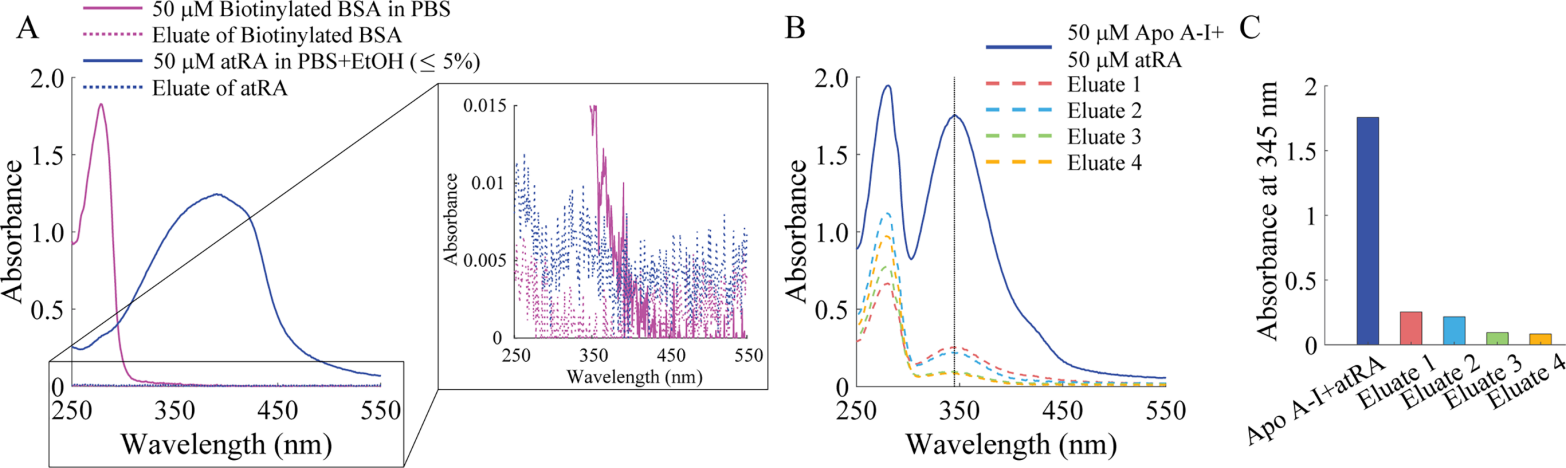
**atRA shows preferential binding to immobilized BSA versus** **soluble Apo A-I.** (**A**) UV-Vis absorbance spectra of separate solutions of biotinylated BSA (50 µM) and atRA (50 µM), and eluates after sequential passage of these solutions through streptavidin resin, showing efficient capture of biotinylated BSA by the resin and atRA by the immobilized BSA. (*Dotted curves* are difficult to visualize as they partially overlap the *horizontal axis*.) For improved clarity, a zoomed in view highlighting the data near the horizontal axis is shown in the inset. (**B**) UV-Vis absorbance spectra of Apo A–I-atRA mixture (solid curve) and eluates after passing through a column bearing immobilized BSA. (**C**) Absorbance intensities of Apo A-I-atRA mixture and subsequent eluates at 345 nm from panel **B**. Panels **A** to **C** show typical results from three technical replicates.

We explored whether atRA would selectively bind to one putative carrier over another in a competition between a surface-immobilized protein and one in solution, behavior that is relevant to the physiological environment where multiple potential carriers are present, some of which may be adherent to extracellular matrix or cells. Biotinylated BSA, immobilized on a column of streptavidin beads, was shown to be able to efficiently capture atRA from solution (Fig. 6A) and to compete with soluble Apo A-I for that same analyte (Figs. 6B, 6C). After several passages of the Apo A-I-atRA mixture through the immobilized BSA, atRA was completely retained on the column and was not dislodged by an additional wash with 10% to 15% ethanol (Supplementary Fig. S12). Control experiments showed no binding of atRA to the bead material (see Supplementary Figs. S12D–S12F). Because the solution-phase association constants of BSA and Apo A-I with atRA are very similar (see the Table), this experiment may highlight the difference between binding to an immobilized protein binding partner (defined by an adsorption coefficient [*K*_ADS_]) and a solution-phase binding partner (defined by association constant [*K*_a_]). For example, in a well-characterized example of carbohydrate-lectin binding, *K*_ADS_ was found to exceed *K*_a_ by a factor of approximately 350.^52^

### floRA Can be Visualized Within Post Mortem Ocular Tissues With Some Limitations

To further investigate floRA's potential utility as a fluorescent tracer within tissue, we exposed mouse choroid/sclera samples to a 50 µM floRA-BSA mixture, allowing the mixture to passively diffuse into the tissue for various lengths of time. Because neither unbound floRA nor BSA fluoresce at 450 to 490 nm in aqueous solution when excited at 340 to 380 nm, any fluorescence we observe in the tissue (above background levels) indicates the presence of protein-floRA complexes.

**Figure 7.**
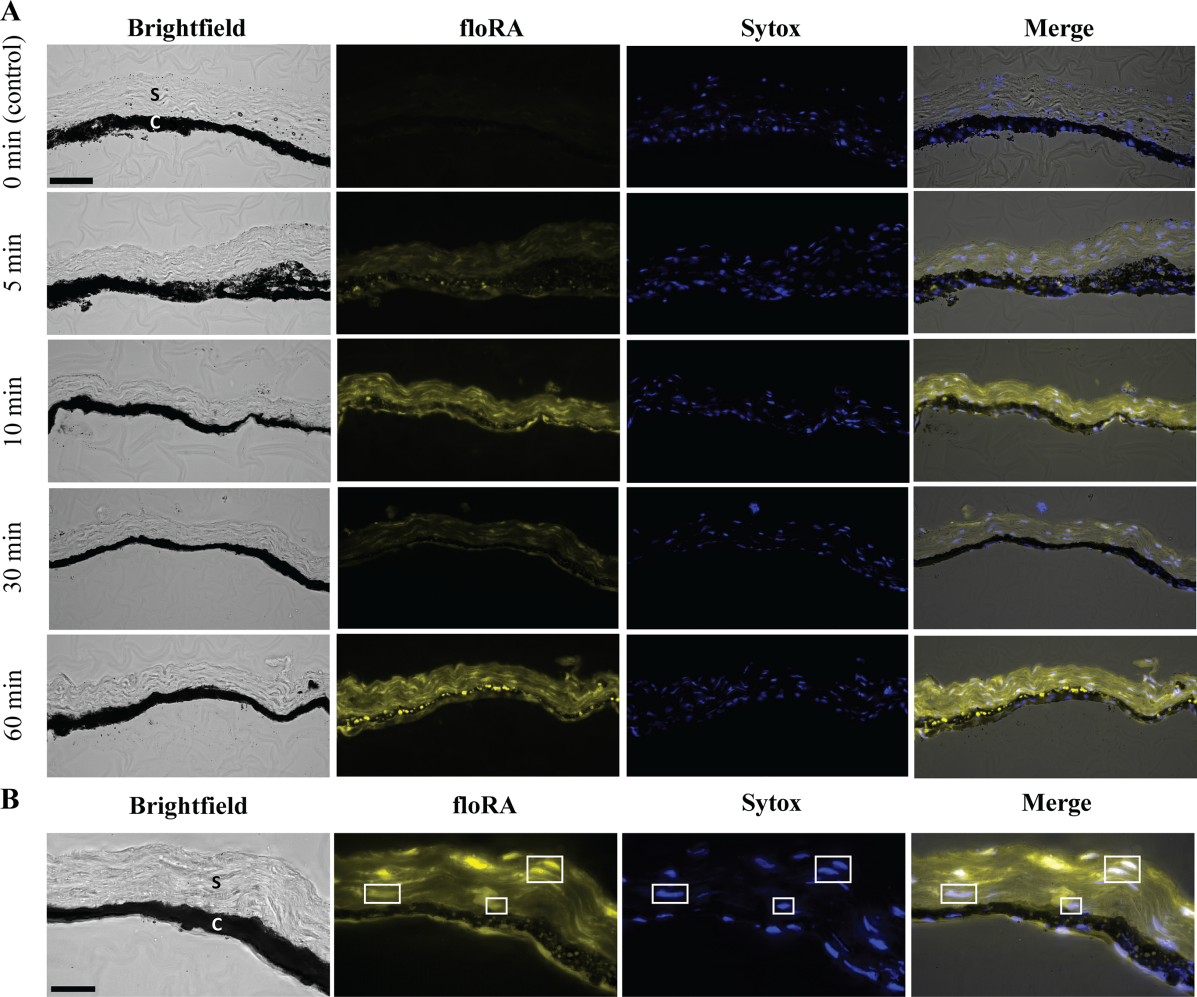
**Incubation of mouse choroid/sclera wedges in a solution of floRA and BSA shows floRA penetration into the tissue.** (**A**) Tissue wedges incubated in floRA-BSA for various durations, including a 0 minute control “incubation.” We show only micrographs up to 60 minutes, because images at later times showed no additional significant features beyond those in this figure. C, choroid; S, sclera; Sytox, nuclear stain. *Scale bar* = 50 µm. (**B**) Visualization of floRA and nuclei showing co-localization, with selected regions *highlighted* in *white boxes*. Abbreviations as in panel **A**. *Scale bar* = 20 µm.

Generally speaking, the amount of fluorescent labeling in both the choroid and sclera increased with increasing incubation duration (Fig. 7A). However, this general trend was non-monotonic, for example, there was markedly less scleral fluorescence at 30 minutes than at 10 minutes in the example shown (Fig. 7A). In a homogeneous material being passively labeled by a diffusive process, one would expect maximum fluorescence at the free surfaces (external margin of the sclera and internal margin of the choroid). However, this was not the case in these experiments: we frequently observed elevated fluorescence in the vicinity of the choroidal-scleral interface, suggesting that the floRA-atRA complex may have rapidly penetrated into the suprachoroidal space in this *post mortem* preparation. We also observed significant spatial heterogeneity, with alternating bright and dark regions within the sclera. Although some of this pattern could be due to tissue deformation (bending) during histological processing, an alternative explanation is that floRA was sequestering within scleral cells. In fact, co-staining for nuclei showed nuclei residing within bright regions of floRA labeling, consistent with this hypothesis (Fig. 7B). It is of interest that a previous study also observed punctate fluorescent structures in HaCaT cells treated with floRA, suggesting the localization of floRA in nuclei and cytoplasmic lipid vesicles.^26^

We attempted to fit the quantitative fluorescence data to a model of unsteady diffusion in a homogenous material,^53^ accounting for the fact that the different scleral samples had different scleral thicknesses and taking the fitted quantity as the spatially averaged concentration of the fluorophore over the entire thickness of the sclera. The fitted quantities were the diffusivity of the floRA-BSA complex within the sclera and the limiting concentration of floRA-BSA in the sclera, i.e. the concentration as incubation time tended to infinity. We assumed that the measured fluorescence was linearly proportional to floRA-BSA concentration. Generally speaking, the fit was poor, for example, taking different starting guesses for the values of the fitted parameters in the nonlinear fitting process produced significantly different outcomes. We conclude that the distribution of fluorescence in the tissue was not well-described by a passive diffusion process in a homogeneous medium. We suggest that the fluctuations in the time series reflect the inherent variability present in these experiments, due to one or more of: (a) spatial tissue heterogeneity; (b) eye-to-eye and mouse-to-mouse variation; and (c) degradation and/or loss of floRA (or its fluorescent response) over the course of the experiment.

## Discussion

We surveyed the binding of several putative carrier proteins with atRA or its synthetic fluorescent analog floRA^26^ to evaluate the potential of using floRA as a surrogate tracer for atRA in studies of myopigenesis, as well as to identify possible atRA carrier proteins within ocular tissues. Our findings indicate that floRA is a reasonable surrogate for atRA binding to BSA, HDL, Apo A-I, and RBP4 due to generally similar binding characteristics. Additionally, whole tissue tracer studies with floRA suggest it can be directly visualized in relevant ocular tissues, but further refinement is required.

### floRA and Other Fluorescent Analogs of atRA

In addition to floRA (LightOx 14), other fluorescent analogs of atRA are available. One such synthetic analog of atRA developed by the same group is EC23,^54^ which exhibits promising photostability and biological activity comparable to that of atRA in cellular differentiation assays. However, its peak excitation wavelength (λ_max_ = 300−310 nm) falls within a range that is damaging to cells, making it less suitable for cellular imaging. To circumvent this limitation, the same group later modified EC23, resulting in significant shifts in its absorption and emission and thus facilitating live cell imaging.^26^ These probes include LightOx 14, 19, 21, 22, 23, 25, and 26 (LightOx Limited, Newcastle upon Tyne, UK) to name a few.

We chose floRA (LightOx 14) over other fluorescent atRA analogs for several reasons:

a. Binding affinity to CRABP2: We used CRABP2 as positive control in our binding affinity experiments,^25^ and thus wanted a fluorescent analog that bound CRABP2 as strongly as possible. Among the commercially available fluorescent analogs, Chisholm et al. previously showed that LightOx 14 and LightOx 19 have the strongest binding affinity with CRABP2.^26^ We thus restricted our attention to LightOx 14 and LightOx 19 over other LightOx derivatives mentioned above.
b. Biological activity: Chisholm et al. further carried out biological characterization of LightOx 14 in human epithelial cells and established that LightOx 14 is able to activate the transcription of the same genes activated by endogenous retinoid atRA and synthetic retinoid EC23.^26^
c. Nuclear localization and imaging feasibility: Chisholm et al. also investigated the localization of LightOx 14 in human keratinocytes, demonstrating that LightOx 14 is detectable within cells using standard fluorescence microscopy and, importantly, exhibits nuclear localization.^26^ This finding suggests that LightOx 14 can interact with retinoic acid transporting proteins, including CRABP2, which shuttles retinoids from cytoplasm to nucleus.

To the best of our knowledge, the functional characterizations described in points b and c were only available for LightOx 14,^26^ which led us to choose LightOx 14 for our study.

### Binding Affinities to Putative Carrier Proteins

atRA and floRA exhibited binding affinity to BSA, HDL, Apo A-I, and RBP4, all proteins that could potentially serve as interstitial retinoic acid chaperones. The observed association constants were found to be very similar, in the range of 2 to 13 × 10^5^ M^−1^ (see the Table; as determined by quadratic model fitting), with the exception of our positive control CRABP2, as expected, and consistent with CRABP2’s role in retinoic acid binding and transport in the cytoplasm.^36^^,^^55^ In biological systems, when multiple proteins are present, the effective atRA transport and carrying capacity of a given protein depends on both the protein’s binding affinity for atRA and its local abundance, and it is therefore of interest to compare relative concentrations of putative carrier proteins.

• In humans, the serum concentration of albumin is typically 35 to 50 mg/mL, whereas in the interstitial space, it is 3- to 5-fold lower.^56^^–^^58^ (We note that this is much larger than the serum concentration of atRA in humans, which typically ranges from 2.8 to 6.6 ng/mL.^59^^,^^60^) In mice, the serum albumin concentration is 10 to 30 mg/mL.^61^
• Typical concentrations of Apo A-I in both human and mouse plasma are 1 to 2 mg/mL^62^^–^^65^ and the average concentration in interstitial fluid is 8- to 12-fold lower.^66^
• In humans, the concentration of retinol-RBP4 in plasma is maintained at 0.04 to 0.06 mg/mL, whereas in mice, it is approximately 0.02 mg/mL.^67^^,^^68^ It is also important to note that whereas the majority (approximately 90%) of RBP4 in the bloodstream exists as retinol-RBP4 complex, a small percentage is also estimated to circulate as free RBP4 (approximately 10%).^69^^–^^71^

In short, Apo A-I and RBP4 concentrations are one or more orders of magnitude lower than corresponding serum albumin concentrations. Considering the similar binding affinities of atRA to various protein binding partners, these data suggest that serum albumin would be expected to be the main binding partner for atRA and floRA transport in the serum^72^ and in the extravascular space in the eye. At first glance, this appears to differ somewhat from the conclusions of Summers, who identified Apo A-I as the dominant atRA-binding protein in the chick eye,^20^ consistent with earlier observations from Mertz and Wallman.^15^ This may reflect a species difference (chicks versus mammals), but it is also important to note that choroidal Apo A-I levels are increased at certain phases of myopigenic recovery and can also be upregulated by atRA.^20^ Future studies of atRA signaling in myopigenesis should therefore consider both albumin and Apo A-I as atRA carriers, as well as assaying the local concentrations of each of these carriers at different phases of myopigenesis/recovery from a myopigenic stimulus.

### Comparison of Protein Binding of atRA Versus floRA

The binding characteristics of atRA and floRA were generally similar (e.g. binding affinities to BSA, HDL, Apo A-I, and RBP4) but also showed some differences (see Supplementary Fig. S5). For example, although we observed floRA-RBP1 binding (K_a_ = 7.9 ± 0.4 × 10^5^ M^−1^; see the Table), it was much weaker than retinol-RBP1 and retinaldehyde-RBP1 binding in which K_a_ falls in the range of a few hundred × 10^5^ M^−1^ as reported in the literature.^29^^,^^30^ RBP1 has a hydrophobic binding pocket optimized for retinol and retinal, which have a terminal hydroxyl (-OH) or aldehyde (-CHO) group, respectively.^73^ atRA has a terminal carboxyl (-COOH) group, which introduces polarity and may cause unfavorable interactions in RBP1’s hydrophobic binding site. floRA, despite being a fluorescent analog of atRA, is not structurally identical to atRA (see Fig. 1) and we speculate that these differences may allow floRA to fit into RBP1’s binding pocket enabling interactions that atRA cannot achieve. Further, we observed that floRA bound somewhat more weakly to RBP4 than did atRA (see Fig. 5, see the Table). The weaker binding between floRA and RBP4 suggests that floRA may not fit optimally within RBP4’s hydrophobic β-barrel binding pocket, which is optimized to specifically host one molecule of retinol.^38^^,^^74^^–^^76^ Thus, although atRA and floRA share important similarities in their affinities for putative binding partners, they are not exact functional equivalents, that is, floRA may not be a good atRA surrogate for transport studies in every circumstance. Further structural investigations are required to distinguish the binding differences between atRA and floRA with several proteins (e.g. RBP1 and RBP4). However, floRA appears to be a reasonable surrogate for determining the location of atRA-BSA complexes within tissue, and thus can be useful for mass transfer tracing studies of atRA in the eye.

### Initial Studies Using floRA as a Tracer in Ocular Tissues

Based on the above, we used floRA-BSA conjugates as a surrogate for visualizing atRA transport in ocular tissues (see Fig. 7). It was possible to observe floRA within ocular tissues but there were limitations with these experiments. These included a tendency for floRA-BSA to preferentially enter the tissue samples via the suprachoroidal space, possibly due to opening of this space during the tissue handling prior to incubation in the floRA-BSA solution. A second concern was the marked spatial heterogeneity observed in the floRA signal within the tissue samples, perhaps due to segregation of floRA within scleral cells, particularly the nuclei. Although this result is encouraging inasmuch it is consistent with the biology of atRA, which exerts its effects in the cell nucleus, it greatly complicates analysis of transport. Future analysis should consider subdividing tissue samples into cellular and acellular zones (based for example on vimentin or other secondary labeling of cells) and quantifying floRA signal in both cellular and acellular regions. A third complication was the pigmentation in the choroid, which possibly interfered with the floRA signal and led us to focus preliminary quantitative analysis on the sclera. In principle, one could circumvent the potential issues with pigment blocking by using an albino mouse strain. However, it is important to note that there are drawbacks to using albino mice, including anatomical (reviewed in Ref. 77) and developmental differences as compared with pigmented strains. For example, the integrity of the retinal pigment epithelium is disrupted in the developing albino mouse.^78^ Additionally, inherent differences in emmetropization mechanisms and varying susceptibility to deprivation myopia have been observed between albino and pigmented guinea pigs.^79^ In view of these differences, we chose to use pigmented animals in this study. Another potential challenge is the limited tissue penetration by shorter wavelengths which may hinder in vivo imaging of posterior ocular structures. During cellular/tissue imaging, floRA fluoresces at 450 to 480 nm when excited at 365 to 405 nm.^26^ Given the relatively thin tissues in the mouse eye, attenuation may be only a modest concern, but experiments in larger eyes could be more challenging. Addressing this limitation will require further research and development. Despite these limitations which precluded rigorous quantitative analysis, our results clearly showed floRA-BSA penetration into both choroid and sclera, consistent with the idea that atRA is an element of the myopigenic retinoscleral signaling pathway.

### Approaches to Measure atRA and Retinoid Levels in Tissue

Previous studies used liquid chromatography tandem mass spectrometry (LC-MS/MS)-based quantification, which is the gold standard for absolute quantification of small molecules,^80^ including atRA.^81^^–^^84^ In addition, Matrix-assisted laser desorption/ionization mass spectrometry imaging (MALDI-MSI) provides spatial localization of metabolites and lipids without the need for physical dissection allowing for the localization of molecules within tissue structures, cell layers and populations, and areas of injury.^85^^–^^87^ Advances have been made toward enabling MALDI-MSI quantification (reviewed in Ref. 88), however, there are still significant limitations including extraction efficiency upon laser desorption, matrix effects, ion suppression, and sensitivity. At present, the requisite sensitivity of MALDI-MSI-based quantification is not sufficient for atRA analysis, with the lowest reported quantitation limits for other molecules being reported at approximately 1 µM,^88^ which is 20 to 30 times greater than typical endogenous levels of atRA in the eye (approximately 10–60 nM).^12^^,^^89^^–^^91^ As the sensitivity of mass spectrometers continues to improve and ion mobility to separate isobaric ions is coupled in hybrid instruments, MSI may be a viable approach for spatial localization of atRA in the future. However, the rigor of LC-MS/MS-based quantification is unlikely to be replaced by MSI approaches and a combination of modalities will be required for elucidating biological mechanisms.

### Limitations

This work is subject to several limitations. For example, the determination of association constants using fluorescence quenching makes some assumptions. In our analysis, we mainly considered static quenching, where the protein and atRA (or floRA) form a nonfluorescent ground state complex. Quenching occurs due to energy transfer from the protein's tryptophan residues to atRA upon complexation. The level of quenching in this scenario depends on the physical proximity of the binding domain and the tryptophan residue. It is possible that protein-ligand complexation takes place, but the binding domain is relatively far from the tryptophan residue(s), thereby impeding the energy transfer. In such a case, interpretation of the fluorescence quenching data as a measure of the actual binding affinity is limited. In addition, the fluorophore (in this case, proteins with intrinsic tryptophan fluorescence) can undergo dynamic quenching through random collision events with the same quencher responsible for static quenching. To overcome these limitations, additional studies using various spectroscopic methods (Fourier-transform infrared, Circular dichroism, fluorescence, etc.)^23^ alongside additional techniques such as microcalorimetry,^92^ and surface plasmon resonance^21^^,^^93^^,^^94^ could be undertaken. Another limitation is that floRA may exhibit different binding and degradation characteristics compared to atRA. We are conducting separate studies to further investigate atRA synthesis and degradation patterns. There may also be differences between in vivo and ex vivo preparations. Given the complexity of atRA transport, it is unlikely that a single experimental method will be sufficient to fully elucidate all aspects of this process. Therefore, we view floRA experiments as one component of a broader, integrated approach that we are actively developing.

A number of species—including macaques, chickens, tree shrews, guinea pigs, marmosets, fish, and mice—have been used to investigate refractive development.^95^ For purposes of this study, we used mice due to their importance in myopia research, due in large part to reagent availability and the capacity for genetic manipulation (extensively reviewed in Ref. 95). This being said, it would of great interest to study floRA transport in other animal models, such as guinea pigs, in future studies.

## Supplementary Material

Supplement 1
