# Supplementary material for: Studies of All-trans Retinoic Acid Transport in the Eye
Source: Invest Ophthalmol Vis Sci. 2025 Jun 30;66(6):84. doi: 10.1167/iovs.66.6.84 (PMC12212452; doi:10.1167/iovs.66.6.84)
Supplement: Supplement 1 [file iovs-66-6-84_s001.pdf]

Supplemental material: Studies of all-*trans* retinoic acid transport in the eye

Saptarshi Chatterjee,[1#] Ankana Roy,[2#] Jianshi Yu,[3] A. Thomas Read,[1] Melissa R Bentley-Ford,[4] Machelle T. Pardue,[1,4,5] Maureen A. Kane,[3] M. G. Finn,[2] C. Ross Ethier[1]

[1]*Wallace H. Coulter Dept. of Biomedical Engineering, Georgia Institute of Technology & Emory University, Atlanta, GA, 30332, USA*
[2] *School of Chemistry and Biochemistry, Atlanta, GA, 30332, USA*
[3]*Department of Pharmaceutical Sciences, University of Maryland, Baltimore, MD, 21201, USA*
[4]*Department of Ophthalmology, Emory University School of Medicine, Atlanta, GA, USA*
[5]*Center for Visual and Neurocognitive Rehabilitation, Atlanta VA Healthcare System, Atlanta, GA, USA*

[#]*These authors contributed equally to this paper.*

Supporting Figures

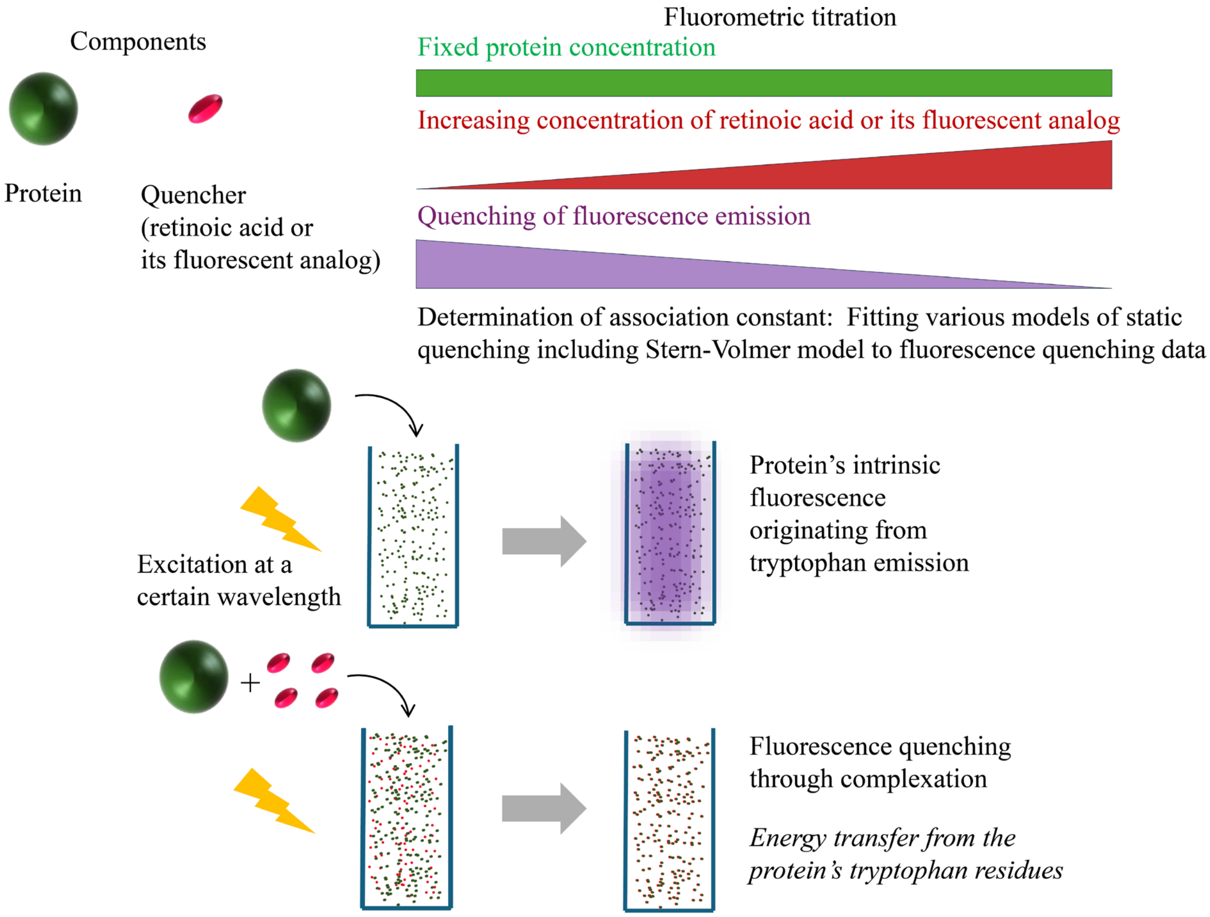

Figure S1 Schematic diagram illustrating the experimental workflow for the fluorescence quenching experiments using carrier proteins and either atRA or its fluorescent analog, floRA.

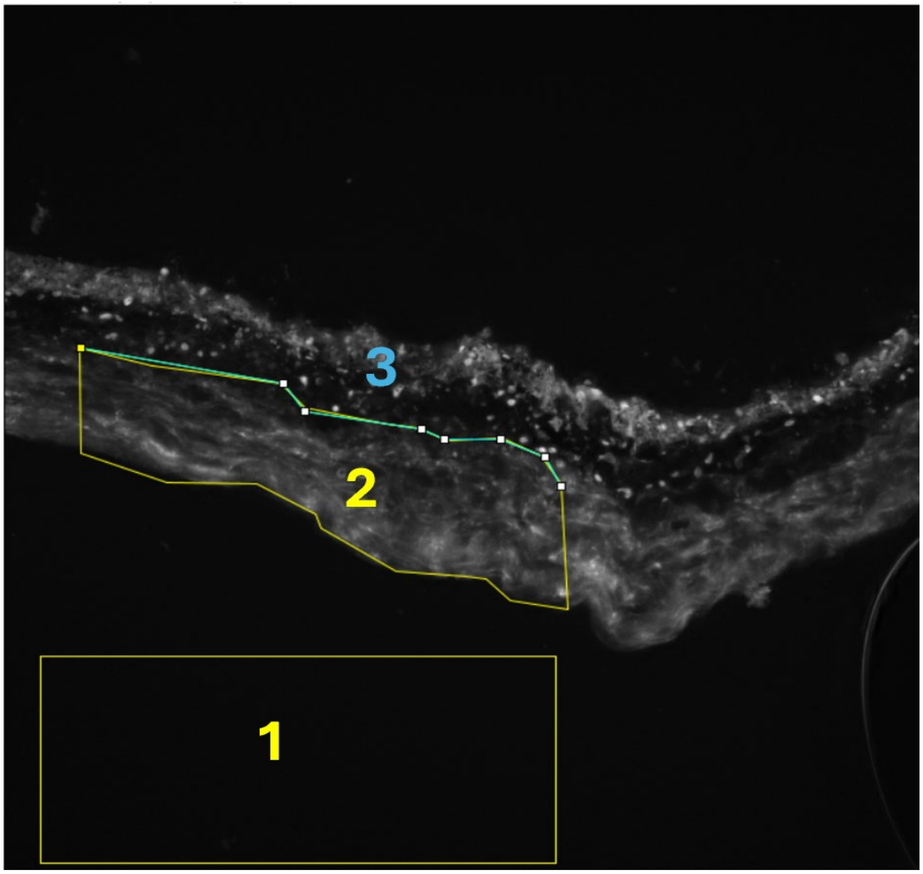

Figure S2: Florescent micrograph showing typical floRA labeling patterns and regions used for quantification of scleral fluorescence intensity. To quantify fluorescence and other metrics we use Fiji to carry out the following steps: (i) Define a rectangle separate from the sectioned tissue (region 1) and determine the mean fluorescence intensity in this rectangle, to be used as the background fluorescence level. (ii) Demarcate a scleral region (region 2) and determine the mean fluorescence intensity (scleral signal) and area of this region. (iii) Extract the length of curve 3, which forms the choroidal-scleral boundary of region 2. From these measurements we compute: (iv) the net fluorescence intensity in the sclera (average fluorescence intensity in region 2 – region 1), and (v) the "thickness" of the sclera, defined as the area of region 2 divided by the length of curve 3. The field of view in this image is 330.2 microns by 330.2 microns.

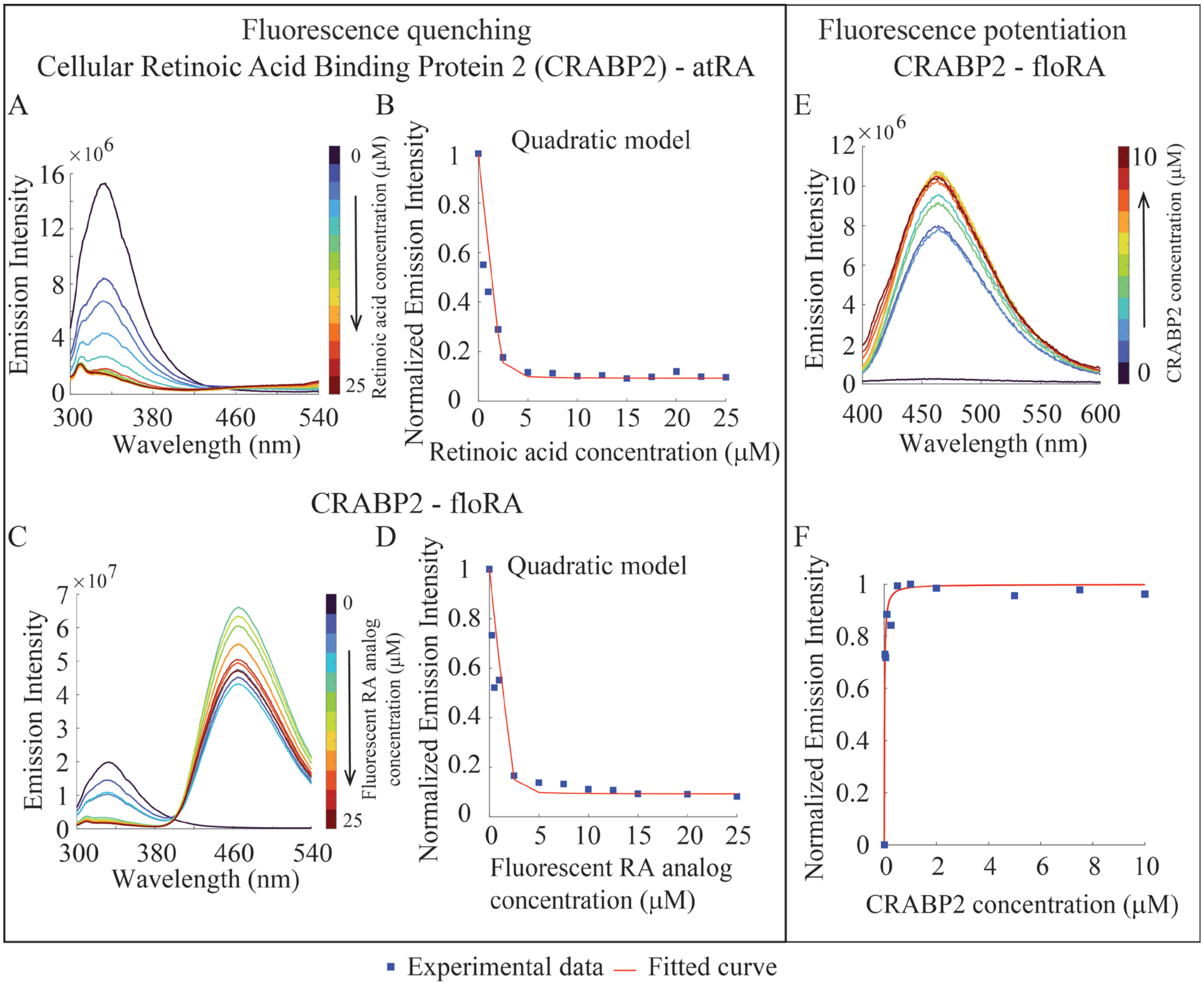

Figure S3 Fluorometric investigations of CRABP2-retinoic acid binding. (A) and **(C)** Fluorescence emission spectra of 2.4 μM CRABP2 in the presence of a range of concentrations (0-25 μM; colored curves) of atRA or floRA, respectively. The quenching of CRABP2 fluorescence with increasing concentrations of atRA or floRA is evident (see peak at 332 nm). There is an additional peak in the spectra in Figure **S3C** due to floRA having an absorbance peak around 280 nm which is absent for atRA (Figure **S4**). **(B)** and **(D)** Quadratic model fits to the peak emission intensities at 332 nm from the spectra in Figure **S3A** and **S3C,** respectively (see Methods). **(E)** Fluorescence emission spectra of 0.05 μM floRA in the presence of a range of concentrations of CRABP2 (0-10 μM; colored curves). With increasing concentration of CRABP2, the fluorescence intensity increases, reflecting the binding of more floRA to CRABP2. **(F)** Fit of the fluorescence emission peaks at 463 nm in Figure **S3E** to a standard Hill curve to estimate the association constant (see Methods). Panels **(A)-(F)** show typical results from 3 technical replicates.

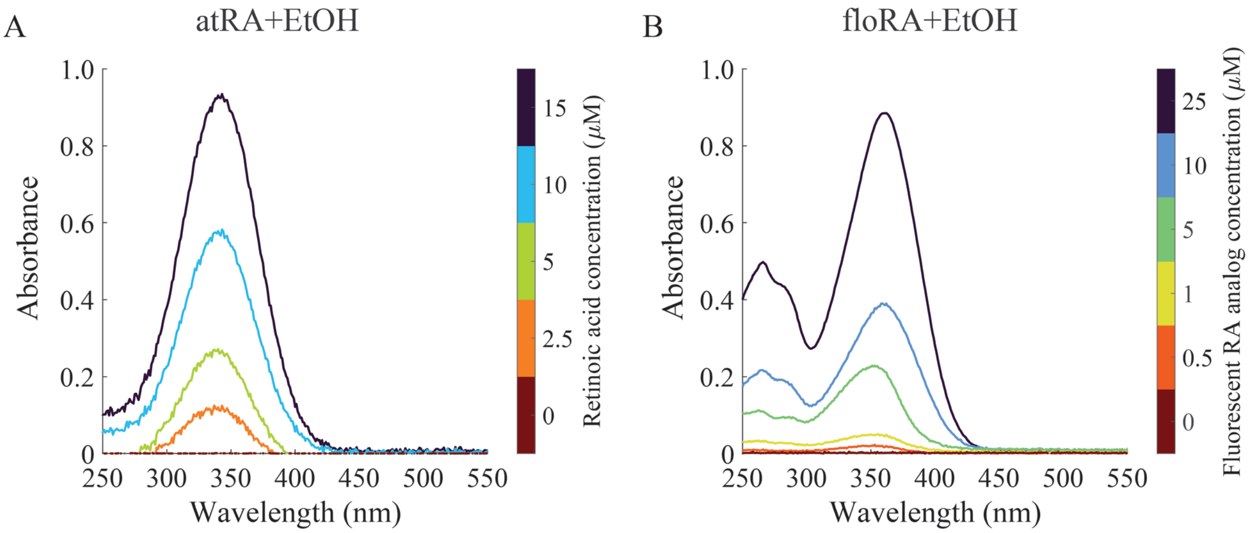

Figure S4 (A) UV-Vis absorbance spectra of 0-15 μM atRA in ethanol, showing an absorption peak around 340 nm **(B)** UV-Vis absorbance spectra of 0-25 μM floRA in ethanol showing absorption peaks around 265 nm, 280 nm, and 360 nm.

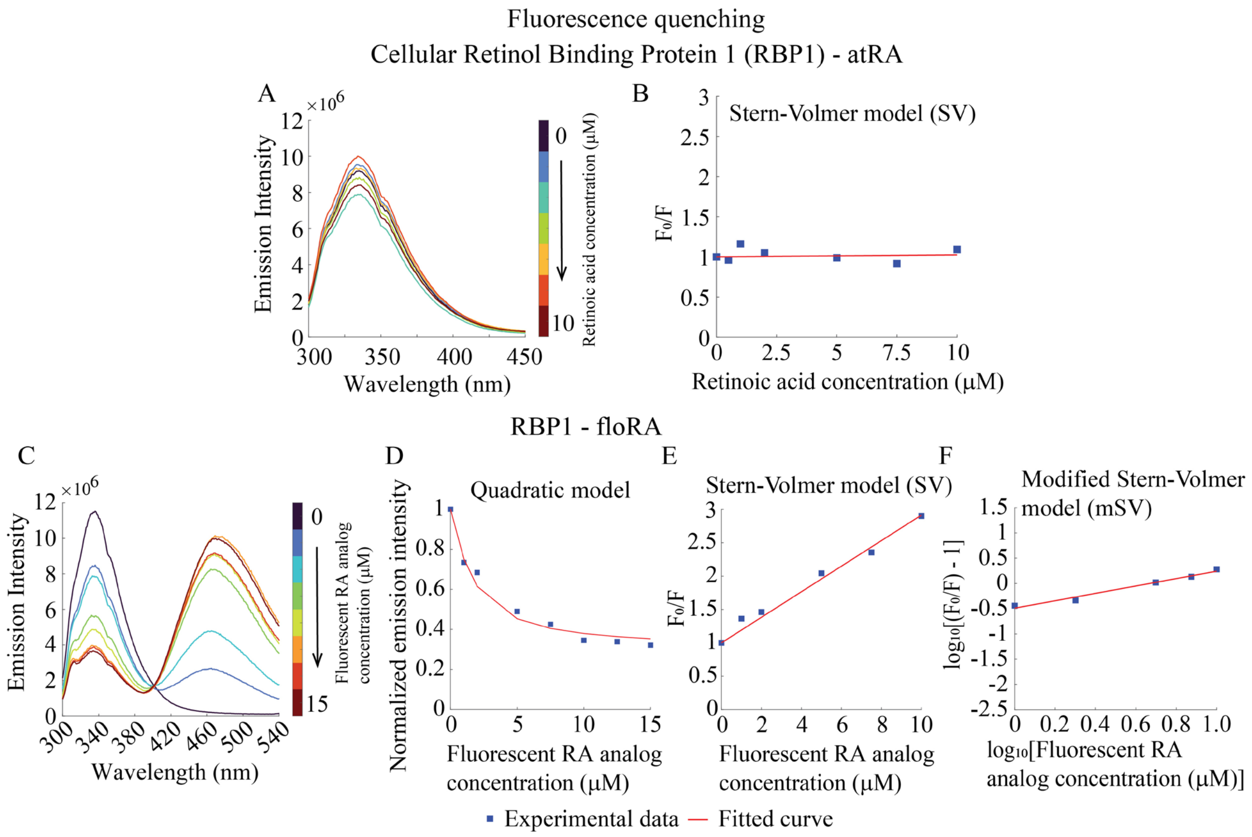

Figure S5 Fluorometric investigations of RBP1-retinoic acid binding. (A) and **(C)** Fluorescence emission spectra of 1.0 μM RBP1 in the presence of a range of concentrations (0-10 μM for atRA and 0-15 μM for floRA; colored curves) of atRA or floRA, respectively. The RBP1 fluorescence does not quench in presence of atRA, but quenches in presence of floRA (see peaks at 334 nm). There is an additional peak in the spectra in Figure **S5C** due to floRA having an

absorbance peak around 280 nm which is absent for atRA (Figure **S4**). **(B)** and **(E)** SV model fits to the peak emission intensities at 334 nm from the spectra in Figure **S5A** and **S5C,** respectively. **(D)** Quadratic model fit to the peak emission intensities at 334 nm from the spectra in Figure **S5C**. **(F)** mSV model fit to the peak emission intensities at 334 nm from the spectra in Figure **S5C**. Panels **(A)**-**(F)** show typical results from 3 technical replicates.

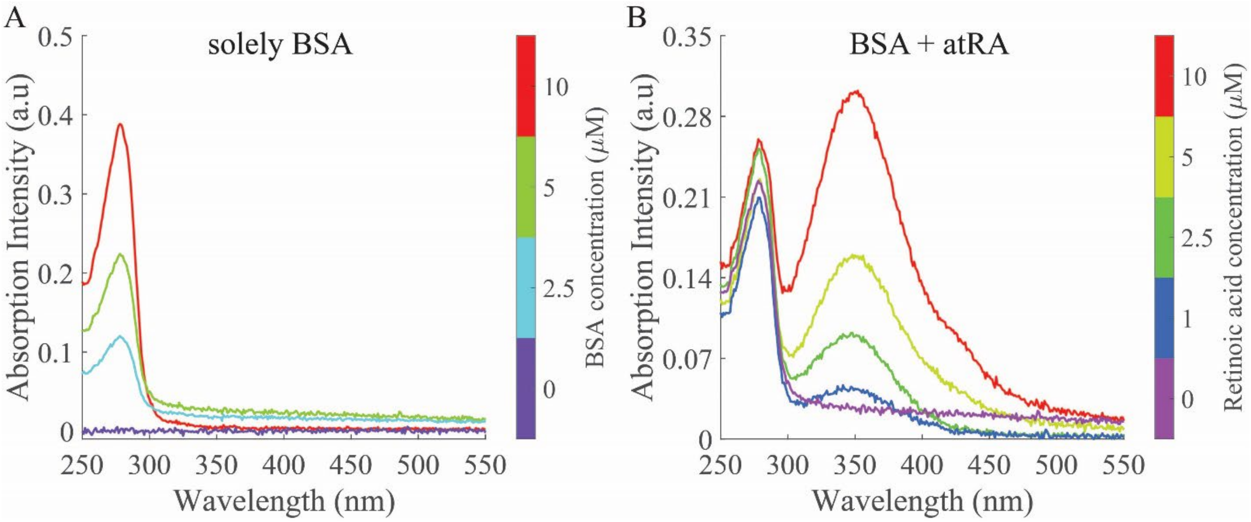

Figure S6 (A) UV-Vis absorbance spectra of 0-10 μM BSA in PBS. The absorbance peak appears around 280 nm **(B)** UV-Vis absorbance spectra of 5 μM BSA in the presence of 0-10 μM atRA in PBS (≤5% EtOH). Absorbance peaks were observed around 280 nm (for BSA) and 350 nm (for atRA).

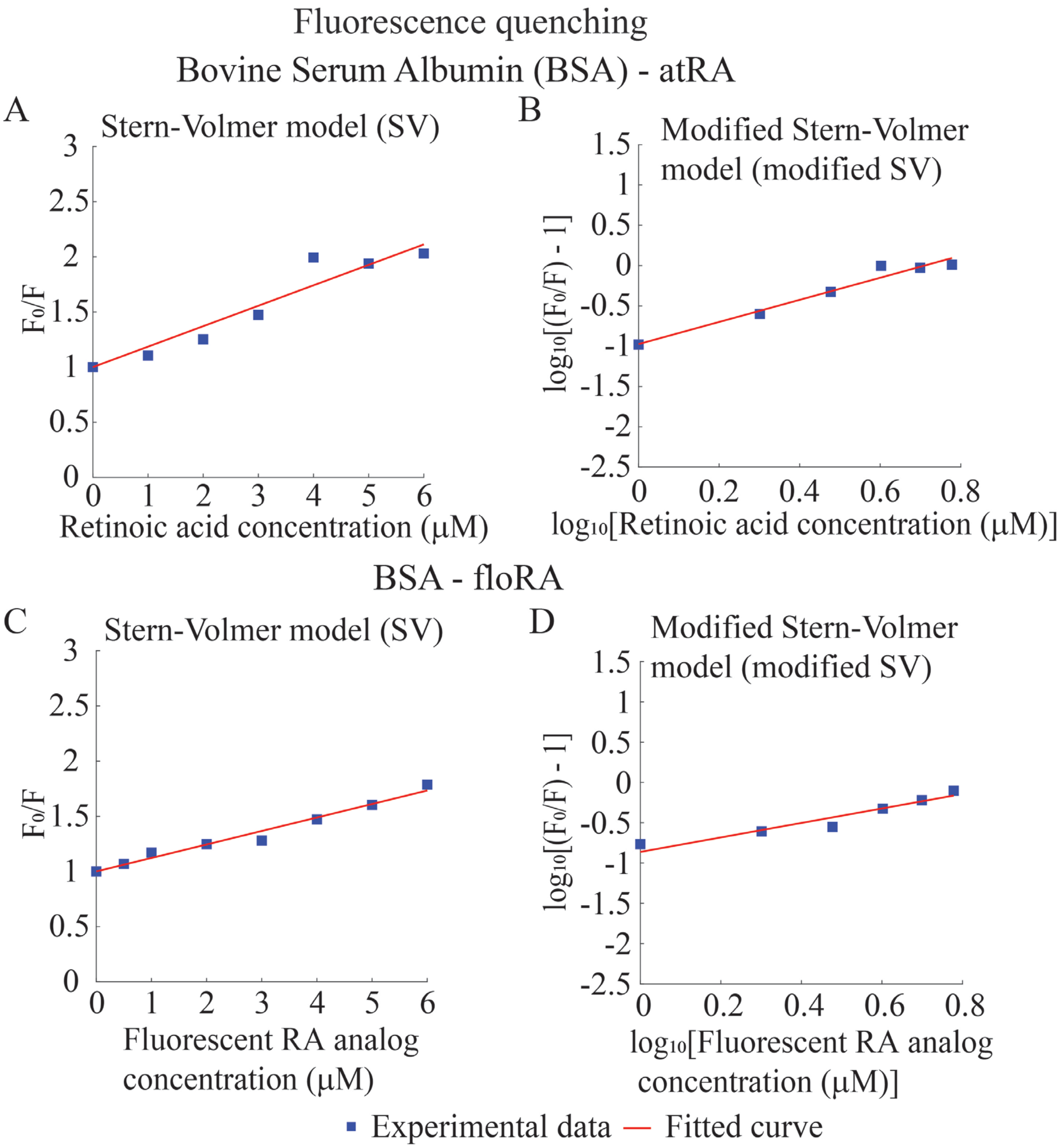

Figure S7 Estimation of association constants for BSA-atRA/floRA complexation (A-B) Stern-Volmer (SV) **(A)** and modified SV **(B)** model fits to the peak emission intensities at 336 nm from the spectra in Figure **2A. (C-D)** SV **(C)** and modified SV **(D)** model fits to the peak emission intensities at 336 nm from the spectra in Figure **2C.**

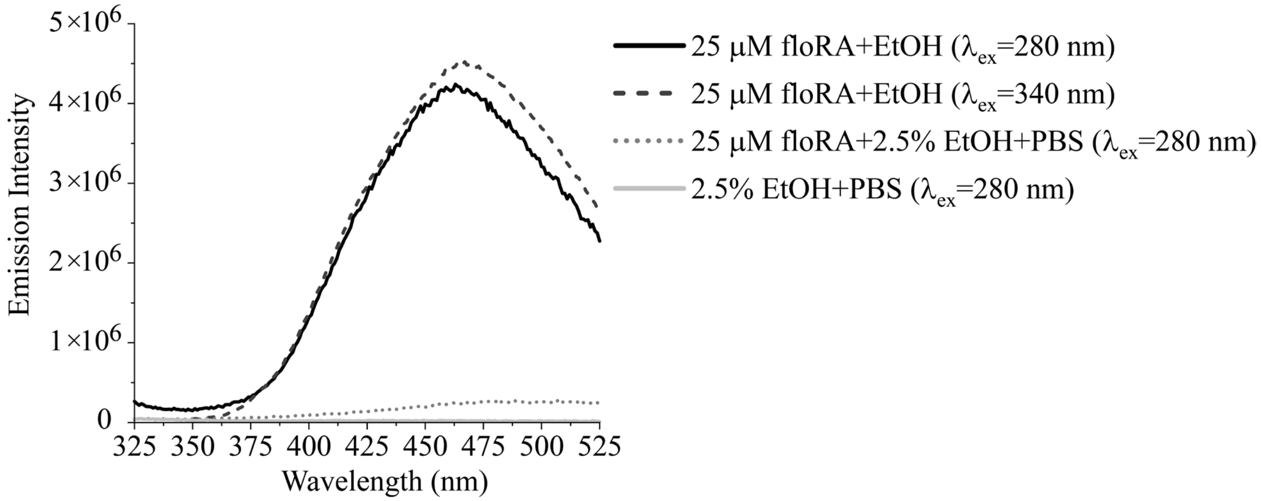

Figure S8 Comparison of the fluorescence spectra of floRA in aqueous vs. non-aqueous solvents. 25 μM floRA shows fluorescence emission in the organic solvent EtOH, but not in aqueous environment (e.g. PBS).

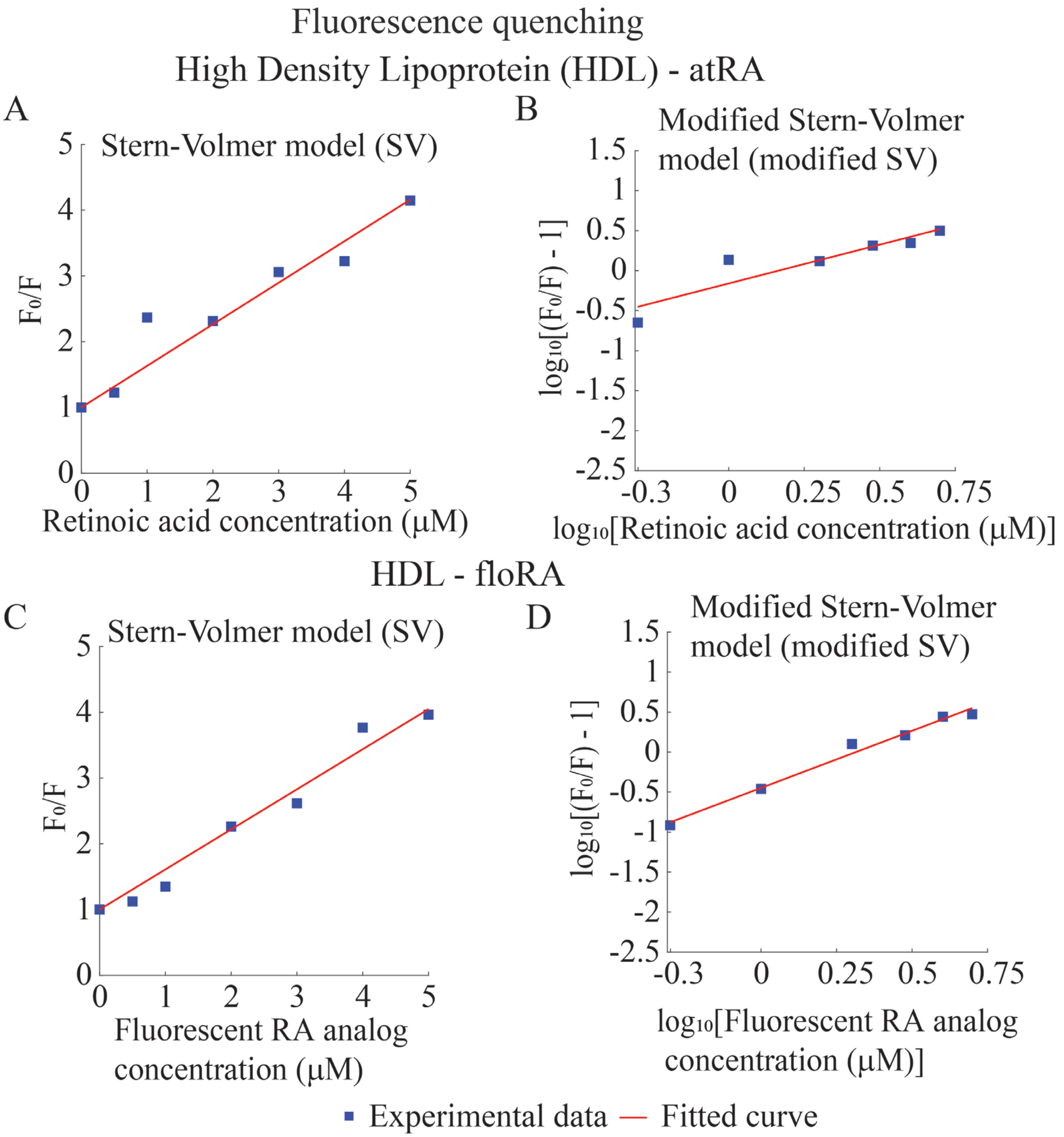

Figure S9 Estimation of association constants for HDL-atRA/floRA complexation (A-B) Stern-Volmer (SV) **(A)** and modified SV **(B)** model fits to the peak emission intensities at 321 nm from the spectra in Figure **3A. (C-D)** SV **(C)** and modified SV **(D)** model fits to the peak emission intensities at 321 nm from the spectra in Figure **3C.** Panels **(A)-(D)** show typical results from 3 technical replicates.

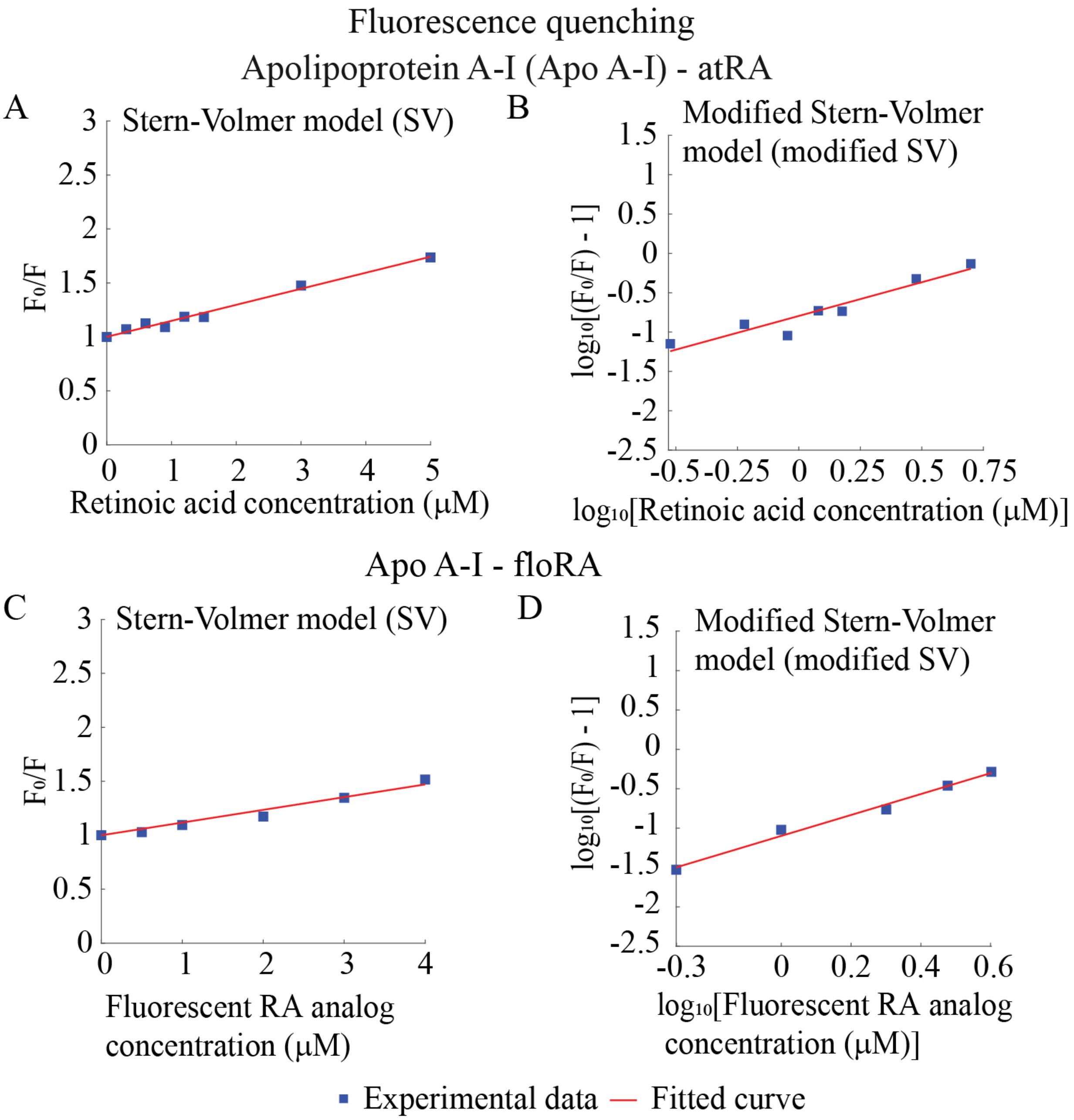

Figure S10 Estimation of association constants for Apo A-I-atRA/floRA complexation (A-B) Stern-Volmer (SV) **(A)** and modified SV **(B)** model fits to the peak emission intensities at 332 nm from the spectra in Figure **4A. (C-D)** SV **(C)** and modified SV **(D)** model fits to the peak emission intensities at 332 nm from the spectra in Figure **4C**. Panels **(A)-(D)** show typical results from 3 technical replicates.

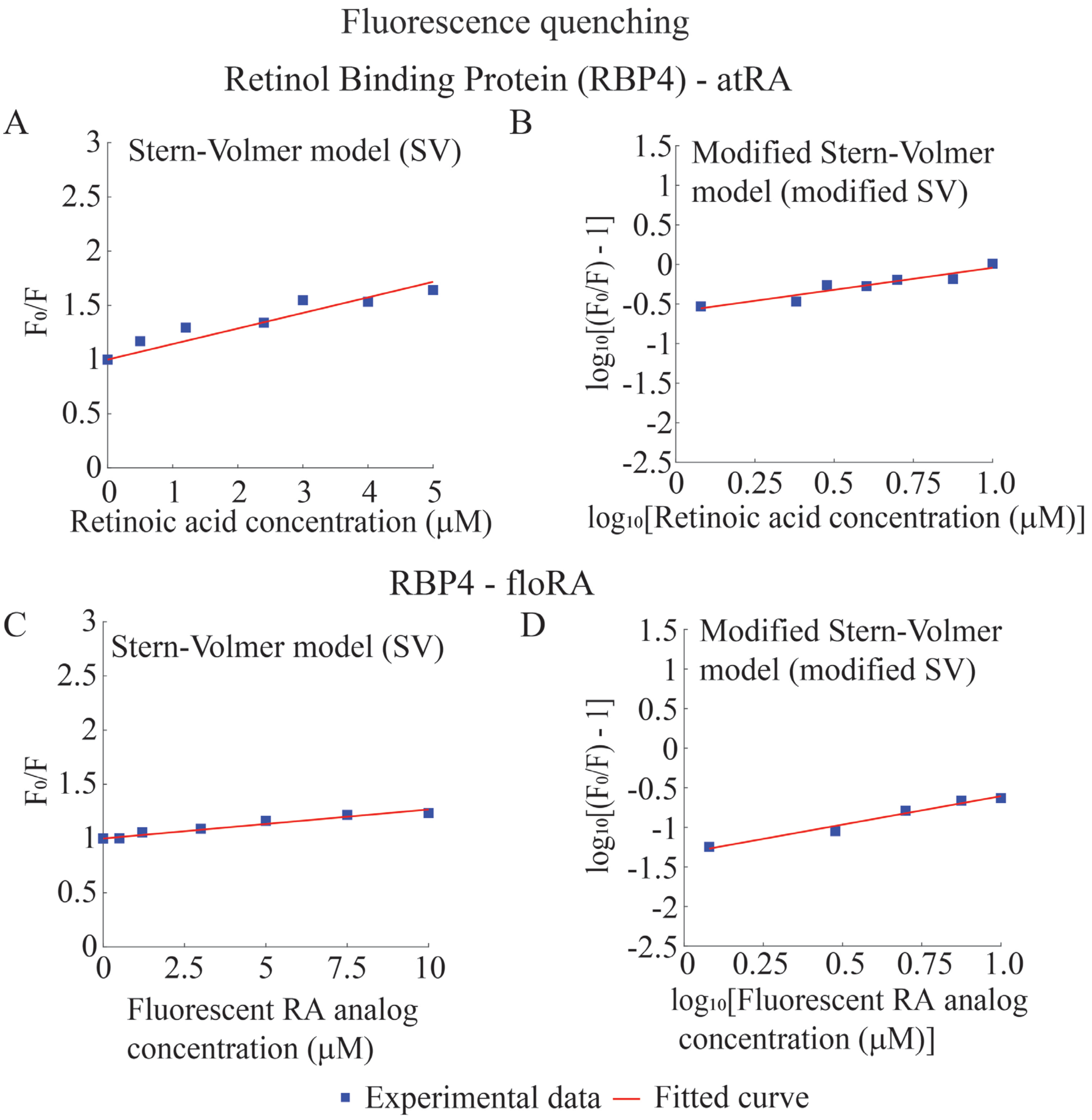

Figure S11 Estimation of association constants for RBP4-atRA/floRA complexation (A-B) Stern-Volmer (SV) **(A)** and modified SV **(B)** model fits to the peak emission intensities at 334 nm from the spectra in Figure **5A. (C-D)** SV **(C)** and modified SV **(D)** model fits to the peak emission intensities at 334 nm from the spectra in Figure **5C.** Panels **(A)-(D)** show typical results from 3 technical replicates.

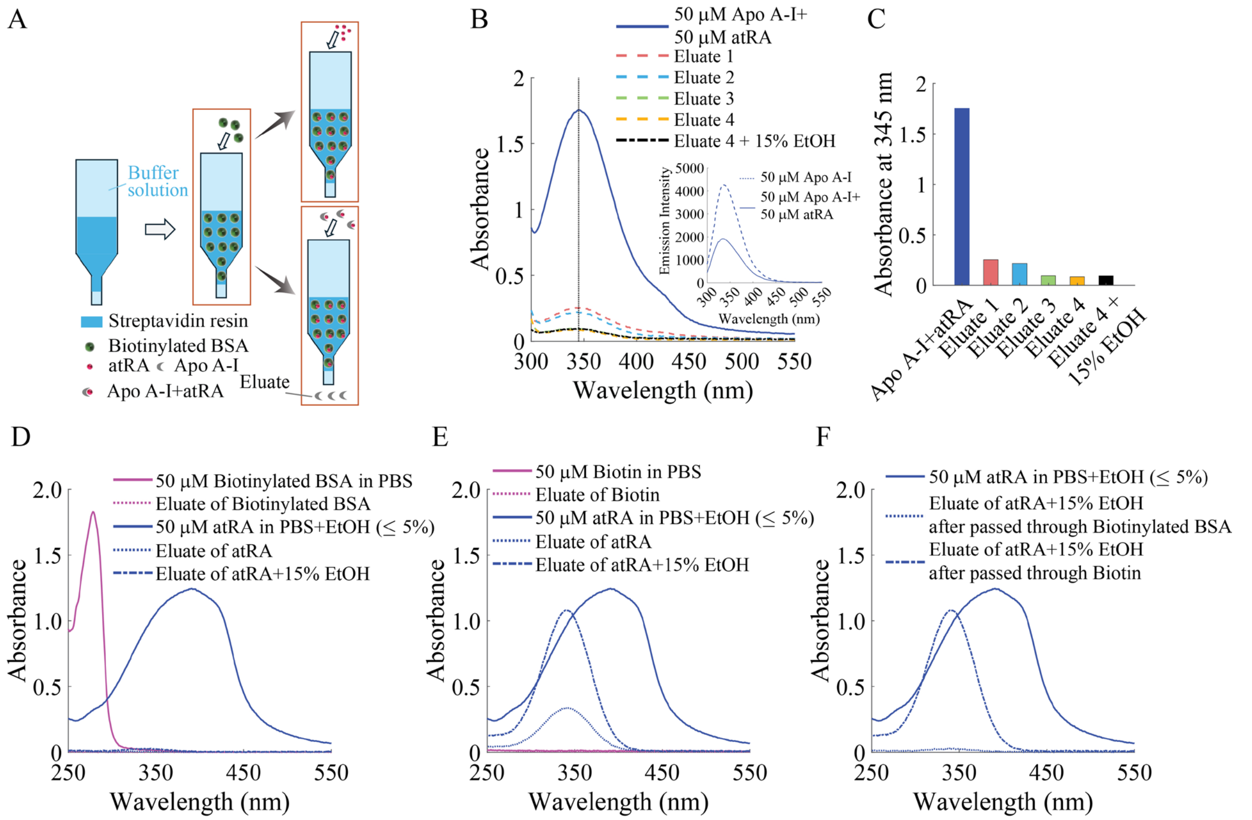

Figure S12 Additional experiments with atRA in presence of biotin, biotinylated BSA and Apo A-I (A) Schematic illustration of the experimental design. Solely atRA and Apo A-I-atRA mixture were separately passed through the biotinylated BSA coated streptavidin resin packed columns. The corresponding eluates were collected for analysis. **(B)** UV-Vis absorbance spectra of Apo A-I-atRA mixture and subsequent eluates. (Inset) A reference emission spectra of 50 μM Apo A-I solution in presence and absence of 50 μM atRA (excitation wavelength 280 nm). (**C**) Absorbance maxima of Apo A-I-atRA mixture and subsequent eluates at 345 nm (as depicted in Figure **S12B**). **(D-F)** UV-Vis absorbance spectra acquired from the control experiments with atRA passed through Biotin/Biotinylated BSA bound streptavidin resin and the corresponding eluates. Some of the dotted/dashed curves are difficult to visualize as they partially overlap the horizontal axis.

Supporting Tables

Protein	Commercial source	Lot no.<br>Catalog no.	Received as
High Density Lipoproteins (HDL), Human Plasma	Athens Research and Technology, GA, USA	HDL2023-09<br>12-16-080412	Liquid; 5 mg in 403 µL of 150 mM NaCl (pH 7.4) 0.01% EDTA
		HDL2023-09<br>12-16-080412	Liquid; 5 mg in 455 µL of 150 mM NaCl (pH 7.4) 0.01% EDTA
Apolipoprotein AI (Apo A-I), Human Plasma	Athens Research and Technology, GA, USA	APOA12022-01<br>16-16-120101	Frozen liquid; 1 mg in 625 µL 10 mM NH_4HCO_3 (pH 7.4)
Retinol Binding Protein (RBP4), Human Plasma	Athens Research and Technology, GA, USA	RBP2020-01<br>16-16-180216	Lyophilized solid.<br>Pre-lyophilized: 100 µg in 39.1 µL 50 mM Tris with 100 mM NaCl (pH 7.4)
Bovine Serum Albumin (BSA)	Fisher Scientific, USA	110466<br>BP1605-100	Refrigerated, lyophilized solid

Table S1 Sources of purchased proteins, and their concentrations as received.

Protein	Ligand	Protein concentration (µM)	Ligand concentration (µM)	Excitation wavelength (nm)	Emission peak (nm) monitored at
BSA	atRA	6	0-25	280	336
	floRA	6	0-25	280	336
HDL	atRA	0.028 mg/ml	0-25	280	321
	floRA	0.028 mg/ml	0-25	280	321
Apo A-I	atRA	1.2	0-25	280	332
	floRA	1.2	0-25	280	332
RBP4	atRA	1.2	0-15	280	334
	floRA	1.2	0-15	280	334
CRABP2	atRA	2.4	0-25	280	332
	floRA	2.4	0-25	280	332
RBP1	atRA	1.0	0-10	280	334
	floRA	1.0	0-15	280	334

Table S2 Experimental details for protein-atRA and protein-floRA fluorescence quenching experiments.

Protein	Ligand	Protein concentration (μM)	Ligand concentration (μM)	Excitation wavelength (nm)	Emission peak monitored (nm)
BSA	floRA	0-25	0.05	340	461
HDL	floRA	0-0.7 mg/ml	0.05	340	437
Apo A-I	floRA	0-35	0.05	340	467
RBP4	floRA	0-18	0.025	340	466
CRABP2	floRA	0-10	0.05	340	463

Table S3 Experimental details for protein-floRA fluorescence potentiation experiments.

Fluorescence Potentiation		
Protein	Ligand	Hill curve fit
		K_a ($\times 10^5$ M^{-1})
BSA	floRA	11.8 ± 1.2
HDL	floRA	10.3 ± 3.5
Apo A-I	floRA	2.5 ± 0.1
RBP4	floRA	2.2 ± 0.5
CRABP2	floRA	539.3 ± 169.6

Table S4 Association constants (mean ± SD) estimated from fluorescence potentiation experiments with floRA and several proteins.

Additional details on the determination of protein-atRA and protein-floRA association constants and stoichiometry from fluorescence quenching data

Stern-Volmer model: The Stern-Volmer model has been widely utilized to evaluate the stoichiometry and binding constants associated with static quenching, where the fluorescently active molecule forms a complex with a quencher molecule.[1-4] In the context of retinoic acid-protein complexation, the protein molecule having tryptophan fluorescence plays the role of the fluorescently active molecule (P) whereas atRA (or floRA) serves as the quencher (Q). This model assumes an equilibrium reaction P + Q ⇌ PQ, with PQ being the nonfluorescent ground state complex. The fluorescence intensity is then expressed in relation to the concentration of the quencher by the classic Stern-Volmer equation[5]:

$$F_0/F = K_a [Q] + 1 \qquad (1)$$

Here F is the fluorescence intensity in the presence of Q, F_0 is the fluorescence intensity in the absence of Q, K_a is the Stern-Volmer constant, and [Q] is the quencher molar concentration, i.e. either atRA or floRA in this study. This model assumes that the quencher forms a complex with the protein and the quenching constant K_a can be considered equivalent to the binding constant of the complexation reaction, as static quenching results from the formation of a non-emissive dark complex between the fluorescently active molecule P and the quencher Q. A modified version of

the Stern-Volmer model considers an equilibrium reaction, $P + nQ \rightleftharpoons PQ_n$, where n units of quencher Q form a complex with a single fluorescently active molecule P. In this scenario, the modified Stern-Volmer equation is:

$$\log_{10}(F_0/F - 1) = \log_{10} K_a + n\log_{10}[Q] \qquad (2)$$

The 'fit' function in MATLAB was used to fit the fluorescence quenching data according to Equation (1) or (2).

Quadratic model: This model considers the same equilibrium reaction, $P + Q \rightleftharpoons PQ$. This model of static quenching, in which quenching occurs through formation of a complex between the fluorescently active molecule and the quencher, assumes 1:1 binding stoichiometry between the protein and the quencher molecules. It also assumes that the quencher (atRA or floRA) is either free in solution or bound to the protein and the fluorescence of the free quencher is zero.[6-8] Considering that the fluorescence quenching is proportional to the degree of binding (ratio of protein-quencher complex and total protein concentration $[P_T]$), the fluorescence emission intensity in a protein-quencher solution can be expressed as:

$$F = F_0 - \frac{1+([P_T]+[Q])K_a - \sqrt{([P_T]-[Q])^2K_a^2+2([P_T]+[Q])K_a + 1}}{2[P_T]\,K_a}(F_0 - F_\infty) \qquad (3)$$

Here, $[P_T]$ is the total protein concentration; [Q] denotes the total quencher concentration (atRA or floRA), K_a denotes the association constant and F_∞ represents the fluorescence signal after the protein has been completely saturated with ligand. This model can deal with nonlinearity in fluorescence quenching, especially at high quencher concentrations, whereas the traditional Stern-Volmer model cannot. To fit Equation (3) to the fluorescence data, we performed a nonlinear least squares curve fitting using MATLAB's 'lsqcurvefit' function.

Additional notes on the analysis of HDL-atRA and HDL-floRA fluorometric titration experiments

The HDL solution we used (Table **S1**) was a mixture of cholesterol, triglyceride and multiple proteins including Apo A-I (molecular mass 28 KDa) and Apo A-II (molecular mass 17.4 kDa), composed of 45-55% lipid with the remainder being proteins. Since this solution was a multi-protein mixture with an unknown fraction of each protein, we report its effective total protein concentration as a mass, rather than a molar concentration. In HDL-atRA quenching experiments (Figure **3** and Figure **S9**), we kept the HDL concentration fixed at 0.028 mg/ml and varied the atRA concentration over the range 0 – 25 μM (Figure **3A-3B**, Table **S2**). However, to fit the quadratic model, we required the molar protein concentration as an input parameter (see the previous section), which was unknown. We therefore treated the effective protein concentration as a free parameter in the model fitting.[6] Using this approach, from HDL-atRA quenching experiments, we obtained an effective protein concentration of 0.73 ± 0.06 μM corresponding to the HDL concentration of 0.028 mg/ml. Of note, a concentration of 0.028 mg/ml Apo A-I (molecular weight 28 kDa) corresponds to approximately 1 μM, aligning closely with the estimated effective protein concentration within HDL. The same approach yielded an effective

protein concentration of approximately 2 μM corresponding to same HDL concentration from the HDL-floRA quenching experiments (Figure **3C-3D**, Table **S2**). In the fluorescence potentiation experiments, the floRA concentration was fixed at 0.05 μM and we varied the HDL concentration over the range 0 – 0.705 mg/ml (Figure **3E-3F**, Table **S3**). Subsequently, we fitted a standard Hill curve to the peak emission intensities monitored at 437 nm (Figure **3F**). We then used the effective protein concentration of 0.028 mg/ml within the HDL solution, which is equivalent to approximately 2 μM (derived from quadratic model fits to HDL-floRA quenching experiments), to estimate the HDL-floRA association constant as $10.3 \pm 3.5 \times 10^5$ M^{-1} (Table **S4**).

References

1. Maiti TK, Ghosh KS, Debnath J, Dasgupta S. Binding of all-trans retinoic acid to human serum albumin: Fluorescence, FT-IR and circular dichroism studies. *Int J Biol Macromol* 2006;38:197–202.
2. N'soukpoe-Kossi CN, Sedaghat-Herati R, Ragi C, Hotchandani S, Tajmir-Riahi HA. Retinol and retinoic acid bind human serum albumin: Stability and structural features. *Int J Biol Macromol* 2007;40:484-490.
3. Belatik A, Hotchandani S, Bariyanga J, Tajmir-Riahi HA. Binding sites of retinol and retinoic acid with serum albumins. *Eur J Med Chem* 2012;48:114-123.
4. Roy A, Das PJ, Diskin-Posner Y, Firer M, Grynszpan F, Montag M. Quenching of syn-bimane fluorescence by Na+ complexation. *New J Chem* 2018;42:15541-15545.
5. Lakowicz JR. *Principles of Fluorescence Spectroscopy*. 3 ed: Springer; 2006.
6. Norris AW, Cheng L, Giguere V, Rosenberger M, Li E. Measurement of subnanomolar retinoic acid binding affinities for cellular retinoic acid binding proteins by fluorometric titration. *Biochim Biophys Acta* 1994;1209:10-18.
7. Kane MA, Bright FV, Napoli JL. Binding affinities of CRBPI and CRBPII for 9-cis-retinoids. *Biochim Biophys Acta* 2011;1810:514-518.
8. Pierzchalski K, Yu J, Norman V, Kane MA. CrbpI regulates mammary retinoic acid homeostasis and the mammary microenvironment. *FASEB J* 2013;27:1904-1916.
